# Supplementary material for: AIRE-Deficient Patients Harbor Unique High-Affinity Disease-Ameliorating Autoantibodies
Source: Cell. 2016 Jul 28;166(3):582–95. doi: 10.1016/j.cell.2016.06.024 (PMC4967814; doi:10.1016/j.cell.2016.06.024)
Supplement: Document S1. Supplemental Experimental Procedures and Tables S1–S4 [file mmc1.pdf]

Cell, Volume *166*

Supplemental Information

AIRE-Deficient Patients Harbor Unique High-Affinity Disease-Ameliorating Autoantibodies

Steffen Meyer, Martin Woodward, Christina Hertel, Philip Vlaicu, Yasmin Haque, Jaanika Kärner, Annalisa Macagno, Shimobi C. Onuoha, Dmytro Fishman, Hedi Peterson, Kaja Metsküla, Raivo Uibo, Kirsi Jäntti, Kati Hokynar, Anette S.B. Wolff, APECED patient collaborative, Kai Krohn, Annamari Ranki, Pärt Peterson, Kai Kisand, and Adrian Hayday

Supplemental Experimental Procedures:

Human samples

Use of human material was approved by local ethics committees (Finland: HUS Medical ERB, 8/13/03/01/2009. Slovenia: National Medical Ethics Committee number 22/09/09 and 28/02/13. Italy: Ethics Committee Prot. PG/2015/20440. Norway: Research Ethics Committee of Western Norway, health registry number 047.96, bio-bank number 2013-1504, project number 2012/1850. Estonia: Research Ethics Committee of the University of Tartu, 235/M-23). All individuals included signed informed consent.

LIPS assay

Coding sequences of IFNs and selected interleukins without signal peptides and *GAD65* were cloned into modified pPK-CMV-F4 fusion vector (PromoCell GmbH, Heidelberg; Germany) downstream of naturally secreted Gaussia luciferase (Gluc), that was cloned into the plasmid instead of Firefly luciferase. HEK 293 cells were transfected with cloned constructs and secreted Gluc-antigen fusion protein was collected with the tissue culture supernatant 48h later. Luciferase immunoprecipitation system (LIPS) assay was modified from Burbelo *et al* (Burbelo et al., 2007). LIPS was performed in 96-well MultiScreen filter HTS plates (Millipore) at room temperature using buffer A (50 mM Tris, pH 7.5, 100 mM NaCl, 5 mM $MgCl_2$, 1% Triton X-100) for all dilutions. IgG from tested samples were captured onto Protein G Agarose beads (25 µl of 4% suspension, Exalpha Biologicals), which were then incubated with supernatantants containing Gluc-antigen fusion protein (10^6 luminescence units (LU) per precipitation reaction). After 1h the plate was washed, Gluc substrate (coelenterazine GAR-2B, Targeting Systems) was injected and luminescence intensity recorded following 1 sec of shaking with the help of Victor X pate reader (PerkinElmer Life Sciences). On average sera from 10-15 control subjects were used as controls in each LIPS experiment.

ELISA

Plates were coated with recombinant human IFNα1 (Immuno Tools), IFNα2 (Immuno Tools), IFNα4 (Sino Biological), IFNα5 (Abnova), IL5 (Immuno Tools), IL6 (Immuno Tools), IL17A (BioLegend), IL17C (BioLegend), IL17F (BioLegend), IL22 (Immuno Tools), IL32α (ImmunoTools) and IL32γ (R&D) with 0.75 µg of protein/ml in PBS, pH 7.0, blocked with 2% HSA. The precoated plates were then incubated with patient serum samples (1:250 dilution) for 2 h at 22 °C, washed, and developed with anti-human IgG horseradish peroxidase conjugate (Sigma-Aldrich), 1-Step Ultra TMB-ELISA (ThermoScientific, Rockford, USA) and Stop Solution (2 M, H_2SO_4). Absorbance was read at 450 nm. The control group used in ELISA consisted of 52 subjects, whereof 43 were healthy relatives of the APECED/APS1 patients (partly overlapping with the samples used in the ProtoArray analysis).

Molecular cloning of comparative recombinant antibodies

The complete Ig-variable heavy- and light chain regions as described in US7741449 (Sifalimumab), US7087726 B2 (Rontalizumab), US8361463 (ACO-1) and US20070258982 A1 (Fezakinumab) were ordered as CHO-codon optimized synthetic constructs (GenScript) and cloned into expression vectors providing the constant regions of human IgG1, human Ig-kappa or human Ig-lambda. The antibodies were produced in HEK293T and CHO cells.

Germline sequences of cloned antibodies

Corresponding closest germlines and heavy chain diversity region sequences were identified based on the nucleotide sequence using the VBASE2 database (Retter et al., 2005). CDRs were identified according to IMGT definitions (Lefranc, 2003). The sequences were ordered, cloned and antibodies expressed as described in previous paragraphs.

EC_{50} ELISA determination of the antibodies

EC_{50} binding of mAbs was determined by ELISA. Serial dilutions of mAbs (from 1000 ng/ml down to 0.0169 ng/ml) were incubated for 2 hours with antigen-coated plates (coating overnight at 1 µg/ml in PBS, followed by wash out and blocking with 2% BSA in PBS). Plates were subsequently washed and binding of mAbs was detected with anti-human HRP-conjugated secondary antibody. Concentrations of mAb resulting in half of maximal binding to respective antigens (EC50, ng/ml) were calculated using Prism 4 GraphPad software on sigmoidal dose-response

curves (variable slope, 4 parameters) obtained by plotting the log of the concentration versus OD_{450} nm measurements.

Phospho-STAT1 assay

30,000 HEK 293T or HEK 293 MSR cells were seeded into Poly-L-Lysine-coated 96-well plates (BD Biocoat, Bedford, MA, USA) or into regular tissue culture-treated 96-well plates (Cat. No. 3598, Corning, Corning, NY, USA), respectively. The following day, recombinant human IFNαs were mixed with anti-IFNα mAbs or control IgG and preincubated for one hour at 37°C. After preincubation, the mixtures were used to stimulate HEK 293T or HEK 293 MSR cells for 10 min at 37°C. Following stimulation, cells were lysed with CelLytic™ M lysis buffer supplemented with protease and phosphatase inhibitors (Cat. No. C2978, P5726, P0044, P8340, SIGMA-ALDRICH, St. Louis, MO, USA) and the collected lysates were cleared at 13,000 RPM, 4°C. Lysates were subjected to reducing SDS-PAGE and blotted onto nitrocellulose membranes. Membranes were blocked with a buffer containing 0.25% bovine gelatin, 150 mM NaCl, 5 mM EDTA, 50 mM Tris/HCl pH 7.5, 0.05% Triton X-100 for one hour at room temperature, followed by incubation with rabbit monoclonal antibodies against phosphorylated STAT1 (Tyr701, diluted in blocking buffer, Cat. No. 9167, Cell Signaling Technology, Danvers, MA, USA) at 4°C over night. On the next day, blots were washed three times with blocking buffer followed by incubation with horseradish peroxidase-linked secondary antibodies against rabbit IgG (diluted in blocking buffer, Cat. No. 111-035-144, Jackson ImmunoResearch, West Grove, PA, USA). After three additional washing steps, an ECL substrate was added (Cat. No. 34087, Thermo Fisher Scientific, Rockford, IL, USA) and reactive bands were visualized via autoradiography. Bound antibodies were removed by incubation in Restore Western Blot Stripping Buffer (Cat. No. 21059, Thermo Fisher Scientific) and a rabbit polyclonal anti-STAT1 serum was used to visualize total STAT1 levels (diluted in blocking buffer, Cat. No. 9172, Cell Signaling Technology). Alternatively, a rabbit monoclonal anti-Tubulin antibody was used to visualize α-Tubulin levels (Cat. No. 2125, Cell Signaling Technology).

ISRE-Luciferase reporter assay

10,000 HEK 293 MSR cells were seeded in white half-area 96-well plates (Cat. No. 3688, Corning) and reverse-transfected with 50 ng of premixed ISRE-Firefly luciferase reporter and Renilla luciferase constructs (Cat. No. CCS-008L, Quiagen, Hilden, Germany) using Fugene HD according to the manufacturer's instructions (Promega, Madison, WI, USA). The Renilla luciferase-expressing construct served as an internal normalization control. Cells were incubated overnight in Opti-MEM® I Reduced Serum Medium supplemented with 0.1 mM non-essential amino acids, 1 mM sodium pyruvate, 0.5% fetal bovine serum (Life Technologies, Carlsbad, CA, USA) at 37°C, 5% CO_2 in a humidified atmosphere. Following overnight incubation, cells were stimulated for 24 hours with medium containing mixtures of recombinant human IFNαs with or without anti-IFNα mAbs or control IgG that had been preincubated for one hour at 37°C. After 24 hours of stimulation, dual luciferase reporter assays were performed according to the manufacturer's instructions (Promega).

Cell-based assay to study Type I IFN neutralizing capacity of patient sera

The IFN neutralizing titer of APS1/APECED sera was tested with the help of reporter cells: HEK-Blue™ IFN- α/β cells (InvivoGen) that express alkaline phosphatase (AP) under the inducible ISG54 promoter after ISGF binding to the IFN-stimulated response elements (ISRE) in the promoter like previously reported (Breivik et al., 2014). The cells were grown in DMEM (Naxo), heat inactivated 10% FBS and supplemented with 30g/ml blasticidin (InvivoGen) and 100g/ml Zeocin (InvivoGen). IFN-α2a (Miltenyi Biotech) was used at final concentration of 12.5 U/ml. IFNα4b and IFNα5 (PBL assay science) were used at final concentration of 37.5 U/ml. IFNAα1, IFNα6, IFNα7, IFNα8, IFNα10, IFNα14, IFNα16, IFNα17, IFNα21, IFNω fusion proteins cloned for LIPS were also used for neutralisation assays. Serial dilutions were made from the antigen preparations to determine the optimal dilution. The dilution that induced approximately similar AP concentration in the stimulated reporter cell supernatant as 3.1 U/ml recombinant IFNα2a was selected for neutralizing assays. Cells were stimulated with optimized concentrations of type I IFNs that were preincubated for 2 hours with serial dilutions of patient or control sera. QUANTI-Blue ™ (InvivoGen) colorimetric enzyme assay was used to determine AP in the cell culture supernatants after 21 hours of incubation. OD was measured at 620nm with Multiscan MCC/340 (Labsystems) ELISA reader. IC_{50} was calculated from the dose-response curves. Statistical analysis of the studied groups (GAD seropositive APS1/APECED patients with (n=8) or without T1D (n=13)) were compared with Mann Whitney test using GraphPad Software (San Diego, CA, USA). Average age were 47.62 ± 11.46 in group with T1D and 31.15 ± 12.14 in the group without T1D.

Other neutralisation assays

Neutralisation assays for IL17F, IL17A/F and IL22 were carried out as previously reported (Kisand et al., 2010).

IL17F neutralisation

NCTC 2544 keratinocytes were pretreated for 3 hours with TNFα (0.1 ng/ml) in DMEM with 10% inactivated FBS. Serial dilutions of mAbs were pre-incubated with 10 ng/ml of IL17F in 96-well culture plate at 37°C. Pre-treated keratinocytes were added after 2 hours at a density of $1x10^4$ cells/well. After incubation at 37°C for 16-20h, supernatants were analysed for growth-related oncogene (GROα) production by ELISA. ED50s are defined as the concentration or titer needed to halve the cytokine activity of the test sample.

IL17A/IL17F heterodimer neutralisation assay

As IL17F neutralisation assay except that mAbs were pre-incubated with 5ng/ml of heterodimer IL17A/IL17F.

IL22 neutralisation

Serial dilutions of mAbs were co-incubated with 0.5 ng/ml of IL22 in 96-well tissue culture plate at 37°C. After 2 hours $3x10^4$ of Colo205 cells in 10% heat-inactivated FBS were added to each well and after 16-20h hours of co-incubation at 37°C, supernatants were collected and analysed for IL10 production by ELISA. ED_{50} values were defined as the concentration or titer needed to halve the cytokine activity of the test sample.

IL20 neutralisation

10,000 HEK 293T MSR cells were seeded in half area white 96-well tissue culture plates (Cat. No. 3688, Corning Inc.). The following day, supernatants of HEK 293T cells transiently expressing human IL20-Gaussia luciferase fusion proteins were mixed with anti-IL-20 mAbs, control IgG or excess concentrations of unlabeled recombinant IL-20 and preincubated for one hour at 37°C. After preincubation, the mixtures were used to stimulate HEK 293T MSR cells transiently expressing IL20 receptors (Type I IL-20 receptor: IL20RA-Myc-DDK (Cat. No. RC212546, OriGene) and IL20RB-Myc-DDK (Cat. No. RC213197); Type II receptor: IL22RA (Cat. No. SC322566, OriGene) and IL20RB-Myc-DDK) for 30 minutes at 37°C. Upon binding, cells were washed three times with PBS, and the gaussia luciferase assay was developed using the Gaussia Flash Assay Kit according to the manufacturer's instructions (Cat. No. 16159, Thermo Fisher Scientific).

IL32 neutralisation

RAW 264.7 macrophages were conditioned in serum-free DMEM overnight. IL-32 (R&D, final concentration 50 ng/ml) was pre-incubated with serial dilutions of serum-free supernatant of HEK293T cells expressing the indicated mAbs in serum-free DMEM for 2 hours at 37° C in a 96-well culture plate. Cells were added at a density of $3x10^4$ cells per well and incubated 18 hours at 37°C in CO_2 incubator. Subsequently, subsequently supernatants were collected and analysed for IL6 production by ELISA. ED_{50} values were defined as the concentration or titer needed to halve the cytokine activity of the test sample.

SPR experiments

A murine mAb directed against human IgG Fc (Human Antibody Capture Kit, GE Healthcare, Piscataway, NJ, USA) was coupled to a CM5 SPR chip (GE Healthcare) using NHS chemistry (Amine Coupling Kit, GE Healthcare) according to the manufacturer's instructions in a Biacore T200 device (GE Healthcare). Human-derived mAbs were immobilized to the coated chip and sensograms were recorded using different concentrations of recombinant human IFN analytes. Data were analysed with Biacore T200 Control Software version 1.0 (GE Healthcare).

Ear inflammation model

All *in vivo* experiments were performed in accordance with relevant institutional and national guidelines and regulations. Ear inflammation phenotype was induced in 8 weeks old C57BL/6J (WT; from Charles River) mice by intradermal injection of human IFNα2a, IFNα2b, IFNα4, IFNα14, IL17F and IL32γ in 20µl of PBS or only PBS (control) into each ear at 2-day intervals starting at day 0 (20µl/ear, 500ng/ear, 1µg total/mouse/day) using a 30-

gauge needle. To test the proinflammatory effect of the injected cytokines, ear thickness measurements of the animals were performed with a Mitutoyo digital micrometer during the cytokine administration, by 2 measurements per ear prior to cytokine injection at day 0 and at alternate days at day 1, day 3, day 5 and alternatively or in addition at day 6 after sacrifice of the animal. RNA was extracted from sacrificed animals and quantitative PCR was performed for TNFα, IFNγ, IL17F, IL22, CD45 and CD3e to measure the level of proinflammatory modulators in affected tissue. Furthermore, body weight was monitored during the treatment, to observe any possible weight changes due to the inflammation induction or its respective reduction due to the treatment applied.

Imiquimod-induced psoriasis-like skin inflammation

C56Bl/6 mice were dorsal back-shaved under anaesthesia 48-72 hours prior to treatment. 24 hours prior to imiquimod application, mice were intra-peritoneally injected with purified antibodies, with further does at 2-day intervals. Control mice were injected with human Ig control. Treated mice received a daily dose of 62.5 mg of commercially available IMQ cream (5%) (Aldera; 3M Pharmaceuticals) for 5 consecutive days. Control mice treated with vaseline. Mice were scored daily using an objective scoring system based on the clinical Psoriasis Area and Severity Index (PASI). Erythema, scaling and thickening were scored independent on a scale form 0to 4: 0 – none, 1 – slight, 2 – moderate, 3 – marked, 4 – very marked. Spleens were weighed, and lymph-node derived T cells were stimulated overnight for analysis by flow cytometry.

Epitope mapping

Overlapping 18mer peptides (14 amino acid overlap) were designed to cover human IL20. The samples were printed onto the microarray slides with a concentration of 1µg/ml. To avoid false negatives caused by steric hindrance, an optimized hydrophilic linker moiety is inserted between the glass surface and the antigen derived peptide sequence. Microarrays were subsequently incubated with the antibodies in blocking buffer for 60 min at 30°C. For detection, an Cy5-anti-human IgG (JIR 209-175-082) directed against the Fc portion of the primary antibody was used at a concentration of 1 µg/ml diluted in blocking buffer and incubated for 60 min at 30°C. Additionally, an incubation with fluorescently labeled secondary antibody only was performed as a control experiment to detect potentially false positives signals. Before each step, microarrays were washed with washing buffer. For analysis, the signal intensities were mapped on the protein sequences to allow identification of linear epitopes. For epitope characterization by Alanine scan a set of 17 peptides comprising the sequence 101-PDHYTLRKISSLANSFL-117 of human IL20 were synthesized. Here, each individual amino acid was separately substituted by Alanine to assess its contribution to the antibody binding.

Table S1. Relating to Figure 2. Frequencies of sero-positive subjects in each cohort

	APS1			Controls/relatives		
**Antigen**	**ProtoArray**	**LIPS**	**ELISA**	**ProtoArray**	**LIPS**	**ELISA**
**IFNα1/13**	87.38%	100.00%	93.33%	4.76%	0.00%	0.00%
**IFNα2**	40.78%	100.00%	90.00%	0.00%	0.00%	0.00%
**IFNα4**	92.23%	100.00%	96.67%	0.00%	0.00%	0.00%
**IFNα5**	68.93%	100.00%	80.00%	0.00%	0.00%	10.00%
**IFNα6**	63.11%	100.00%	n.d.	0.00%	0.00%	n.d.
**IFNα7**	n.d.	100.00%	n.d.	n.d.	0.00%	n.d.
**IFNα8**	57.28%	100.00%	n.d.	0.00%	0.00%	n.d.
**IFNα10**	n.d.	100.00%	n.d.	n.d.	0.00%	n.d.
**IFNα14**	70.87%	100.00%	n.d.	0.00%	0.00%	n.d.
**IFNα16**	23.30%	100.00%	n.d.	0.00%	0.00%	n.d.
**IFNα17**	58.25%	100.00%	n.d.	0.00%	0.00%	n.d.
**IFNα21**	44.66%	100.00%	n.d.	0.00%	0.00%	n.d.
**IFNω**	44.66%	100.00%	25.37%	0.00%	0.00%	0.00%
**IFNκ**	0.00%	0.00%	n.d.	0.00%	0.00%	n.d.
**IFNγ**	0.97%	0.00%	0.00%	0.00%	0.00%	0.00%
**IFNβ**	n.d.	12.50%	n.d.	0.00%	0.00%	n.d.

	APS1			Controls/relatives		
**Antigen**	**ProtoArray**	**LIPS**	**ELISA**	**ProtoArray**	**LIPS**	**ELISA**
**IL1A**	1.94%	21.25%	n.d.	0.00%	0.00%	n.d.
**IL4**	0.00%	0.00%	0.00%	0.00%	0.00%	0.00%
**IL5**	2.91%	16.25%	4.48%	0.00%	0.00%	0.00%
**IL6**	n.d.	27.63%	8.96%	n.d.	0.00%	0.00%
**IL9**	0.00%	0.00%	0.00%	0.00%	0.00%	0.00%
**IL17A**	18.45%	35.00%	5.97%	0.00%	0.00%	0.00%
**IL17C**	3.88%	0.00%	22.39%	0.00%	0.00%	0.00%
**IL17F**	0.00%	70.00%	31.34%	0.00%	0.00%	0.00%
**IL20**	0.00%	8.75%	0.00%	0.00%	0.00%	0.00%
**IL21**	0.97%	0.00%	0.00%	0.00%	0.00%	0.00%
**IL22**	0.00%	93.75%	37.31%	0.00%	0.00%	0.00%
**IL28**	n.d.	28.00%	0.00%	n.d.	0.00%	0.00%
**IL29**	n.d.	53.75%	0.00%	n.d.	0.00%	0.00%
**IL32[illegible]**	n.d.	n.d.	7.46%	n.d.	n.d.	1.82%
**IL32γ**	5.83%	n.d.	6.67%	0.00%	0.00%	0.00%
**G-CSF**	0.00%	0.00%	n.d.	0.00%	0.00%	n.d.
**GM-CSF**	0.00%	0.00%	n.d.	0.00%	0.00%	n.d.

Samples were considered positive when *Z* scores ≥3 in ProtoArray, relative luciferase units ≥3 in LIPS and OD_{450} ≥1.5 in ELISA. n.d. not determined.

Table S2. Relating to Figure 3. Binding and neutralisation of Type I IFNs

	5D1		13B11		19D11		25C3		26B9		31B4	
**Human**	B	N	B	N	B	N	B	N	B	N	B	N
**IFNα1/13**	-	-	++	++	++	++	++	++	++	++	++	++
**IFNα2**	++	++	++	++	++	++	++	++	++	++	++	++
**IFNα4**	++	++	++	++	++	++	++	++	++	++	++	++
**IFNα5**	++	++	++	++	++	++	++	++	++	++	++	++
**IFNα6**	++	+	+	-	++	+	++	+	++	++	++	++
**IFNα8**	++	++	-	-	++	++	++	+	++	++	++	++
**IFNα14**	+	-	++	++	++	++	++	+	++	++	++	++
**IFNα16**	*nd*	++	*nd*	++	*nd*	++	*nd*	+	*nd*	-	*nd*	-
**IFNα21**	++	++	-	-	+	++	++	++	++	++	++	++
**Mouse**												
**IFNα2**	+/-	*nd*	-	*nd*	+/-	*nd*	+	*nd*	-	*nd*	-	*nd*
**IFNα4**	-	*nd*	-	*nd*	-	*nd*	-	*nd*	-	*nd*	-	*nd*
**IFNα14**	-	*nd*	-	*nd*	+/-	*nd*	-	*nd*	-	*nd*	-	*nd*

B: Binding as determined by ELISA. N: Neutralisation as determined by ISRE luciferase reporter assay or by STAT phosphorylation assessed by western blot.
+: Positive
-: Negative
nd: not determined

Table S3. Relating to Figure 3. *In vitro* characteristics of APECED/APS1 derived-patient antibodies

		Antibody ID		
**IL17**		**24D3**	**17E3**	**9A2**
	Isotype	IgG1, λ	IgG1, k	IgG1, k
	EC_{50} (ng/ml)	IL17F: 2.64<br>IL17A: -	IL17F: 2<br>IL17A: 824	IL17F: 2.78<br>IL17A: -
	IC_{50} (ng/ml)	IL17F: 12-22<br>IL17A/F: 6-14	IL17F: 15<br>IL17A/F: 12	IL17F: 12.5<br>IL17A/F: -
	Affinity (SPR)	<10 pM	<10 pM	n.d.
**IL22**		**30G1**	**35G11**	**Fezakinumab**
	Isotype	IgG1, k	IgG4, λ	IgG1, λ
	EC_{50} (ng/ml)	Human: 3<br>Mouse: 15	Human: 7<br>Mouse: n.c.*	Human: 44<br>Mouse: +**
	IC_{50} (ng/ml)	Human: 2.1<br>Mouse: 1.5	Human: 3.8<br>Mouse: n.c.*	Human: +**<br>Mouse: +**
	Affinity (SPR)	37 pM	39 pM	54 pM**
**IL32γ**		**2C2**		
	Isotype	IgG3, λ		
	EC_{50} (ng/ml)	400		
	IC_{50} (ng/ml)	300		
	Affinity (SPR)	~4nM		
**IL20**		**20A10**	**2A11**	
	Isotype	IgG4, k	IgG1, λ	
	EC_{50} (ng/ml)	58.3±21	93 ± 34	
	IC_{50} (ng/ml)	Type I: 4.03<br>Type II: 6.26	Type I: 13.2<br>Type II: 70.7	
	Affinity (SPR)	91 fM	n.d.	

n.d.: not determined

Table S4. Relating to Figure 4. Neutralisation of Type I IFNs

	IC_{50} (ng/ml)			
**Antigen**	19D11	26B9	Sifalimumab	Rontalizumab
IFNα1	3.80	8.60	460.0	22.63
IFNα2	1.62	2.83	9.02	2.15
IFNα4	0.95	2.07	35.35	325.3
IFNα5	0.85	3.74	93.29	2.49
IFNα6	0.79	3.16	4.97	-
IFNα7	0.37	1.57	233.1	-
IFNα8	27.69	205.0	691.0	10.86
IFNα10	0.72	1.84	43.20	-
IFNα14	0.31	2.01	14.52	0.90
IFNα16	1.86	4013.0	59.18	28.65
IFNα17	0.75	2.22	890.9	23.86
IFNα21	2.22	4.65	1769.0	5.80
IFNω	-	0.50	-	-

Supplemental References

Breivik, L., Oftedal, B.E., Boe Wolff, A.S., Bratland, E., Orlova, E.M., and Husebye, E.S. (2014). A novel cell-based assay for measuring neutralizing autoantibodies against type I interferons in patients with autoimmune polyendocrine syndrome type 1. Clin. Immunol *153*, 220-227.

Burbelo, P.D., Ching, K.H., Mattson, T.L., Light, J.S., Bishop, L.R., and Kovacs, J.A. (2007). Rapid antibody quantification and generation of whole proteome antibody response profiles using LIPS (luciferase immunoprecipitation systems). Biochem. Biophys. Res. Commun *352*, 889-895.

Kisand, K., Boe Wolff, A.S., Podkrajsek, K.T., Tserel, L., Link, M., Kisand, K.V., Ersvaer, E., Perheentupa, J., Erichsen, M.M., Bratanic, N., *et al.* (2010). Chronic mucocutaneous candidiasis in APECED or thymoma patients correlates with autoimmunity to Th17-associated cytokines. J. Exp. Med *207*, 299-308.

Lefranc, M.P. (2003). IMGT, the international ImMunoGeneTics database. Nucleic Acids Res *31*, 307-310.

Retter, I., Althaus, H.H., Munch, R., and Muller, W. (2005). VBASE2, an integrative V gene database. Nucleic Acids Res *33*, D671-674.

Sboner, A., Karpikov, A., Chen, G., Smith, M., Mattoon, D., Freeman-Cook, L., Schweitzer, B., and Gerstein, M.B. (2009). Robust-linear-model normalization to reduce technical variability in functional protein microarrays. J. Proteome Res *8*, 5451-5464.
