# Supplementary material for: AIRE-Deficient Patients Harbor Unique High-Affinity Disease-Ameliorating Autoantibodies
Source: Cell. 2016 Jul 28;166(3):582–95. doi: 10.1016/j.cell.2016.06.024 (PMC4967814; doi:10.1016/j.cell.2016.06.024)
Supplement: Document S2. Article plus Supplemental Information [file mmc2.pdf]

Cell

Article

AIRE-Deficient Patients Harbor Unique High-Affinity Disease-Ameliorating Autoantibodies

Graphical Abstract

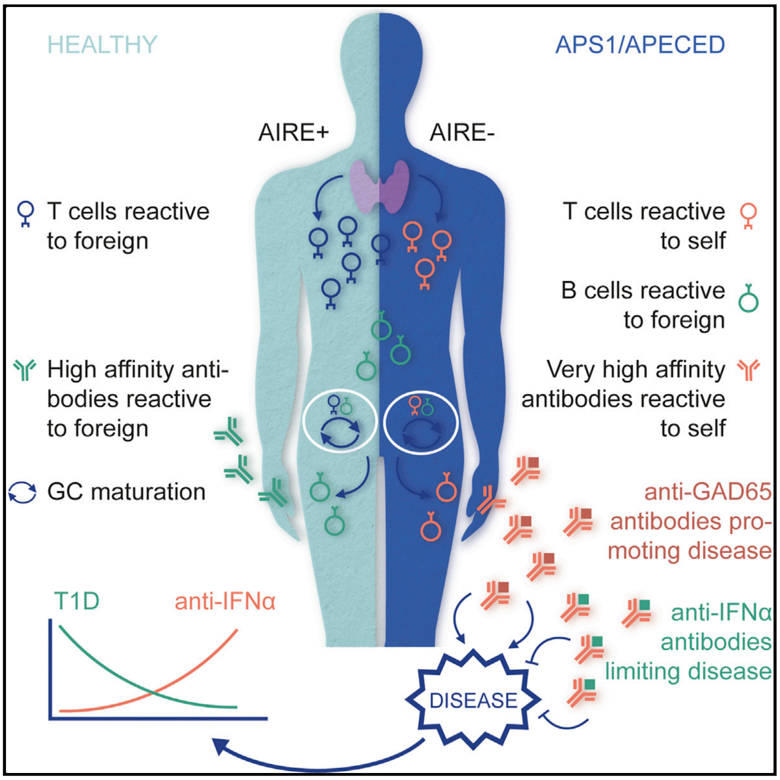

Highlights

- Each AIRE-deficient patient has a private repertoire of autoantibody reactivities
- Loss of B cell tolerance occurs during T cell-dependent somatic hypermutation
- Patient autoantibodies have unprecedented affinities for conformational epitopes
- Patient autoantibodies can display disease-ameliorating properties in vivo

Authors

Steffen Meyer, Martin Woodward, Christina Hertel, ..., Pärt Peterson, Kai Kisand, Adrian Hayday

Correspondence

kai.kisand@ut.ee (K.K.),
adrian.hayday@kcl.ac.uk (A.H.)

In Brief

Self-reactive antibodies specific for type I interferons are associated with protection against type I diabetes in patients with an autoimmune syndrome caused by mutations in AIRE.

Meyer et al., 2016, Cell *166*, 582–595
July 28, 2016 © 2016 The Authors. Published by Elsevier Inc.
http://dx.doi.org/10.1016/j.cell.2016.06.024

CellPress

AIRE-Deficient Patients Harbor Unique High-Affinity Disease-Ameliorating Autoantibodies

Steffen Meyer,[1,11] Martin Woodward,[2,11] Christina Hertel,[1,11] Philip Vlaicu,[1,11] Yasmin Haque,[2,11] Jaanika Kärner,[3,11] Annalisa Macagno,[4] Shimobi C. Onuoha,[4] Dmytro Fishman,[5,6] Hedi Peterson,[5,6] Kaja Metsküla,[7] Raivo Uibo,[7] Kirsi Jäntti,[8] Kati Hokynar,[8] Anette S.B. Wolff,[9] APECED patient collaborative, Kai Krohn,[8] Annamari Ranki,[10] Pärt Peterson,[3] Kai Kisand,[3,*] and Adrian Hayday[2,*]

[1]ImmunoQure AG, Königsallee 90, 2012 Düsseldorf, Germany
[2]Peter Gorer Department of Immunobiology, King's College, London SE19RT, UK
[3]Molecular Pathology, Institute of Biomedicine and Translational Medicine, University of Tartu, Ravila 19, Tartu 50411, Estonia
[4]ImmunoQure Research AG, Wagistrasse 14, 8952 Schlieren, Switzerland
[5]Institute of Computer Science, University of Tartu, Liivi 2, Tartu 50409, Estonia
[6]Quretec Ltd., Ülikooli 6A, Tartu 51003, Estonia
[7]Department of Immunology, Institute of Biomedicine and Translational Medicine, University of Tartu, Ravila 19, Tartu 50411, Estonia
[8]Clinical Research Institute HUCH Ltd., Haartmaninkatu 8, 00290 Helsinki, Finland
[9]Department of Clinical Science, University of Bergen, Laboratory Building, 8th floor, 5021 Bergen, Norway
[10]Department of Dermatology, Allergology and Venereology, Institute of Clinical Medicine, University of Helsinki, Skin and Allergy Hospital, Helsinki University Central Hospital, Meilahdentie 2, 00250 Helsinki, Finland
[11]Co-first author
*Correspondence: kai.kisand@ut.ee (K.K.), adrian.hayday@kcl.ac.uk (A.H.)
http://dx.doi.org/10.1016/j.cell.2016.06.024

SUMMARY

APS1/APECED patients are defined by defects in the autoimmune regulator (AIRE) that mediates central T cell tolerance to many self-antigens. AIRE deficiency also affects B cell tolerance, but this is incompletely understood. Here we show that most APS1/APECED patients displayed B cell autoreactivity toward unique sets of approximately 100 self-proteins. Thereby, autoantibodies from 81 patients collectively detected many thousands of human proteins. The loss of B cell tolerance seemingly occurred during antibody affinity maturation, an obligatorily T cell-dependent step. Consistent with this, many APS1/APECED patients harbored extremely high-affinity, neutralizing autoantibodies, particularly against specific cytokines. Such antibodies were biologically active in vitro and in vivo, and those neutralizing type I interferons (IFNs) showed a striking inverse correlation with type I diabetes, not shown by other anti-cytokine antibodies. Thus, naturally occurring human autoantibodies may actively limit disease and be of therapeutic utility.

INTRODUCTION

T lymphocyte tolerance is essential for limiting autoimmune disease. Tolerance occurs "centrally" when developing thymocytes with strongly self-reactive T cell receptors (TCRs) are deleted following engagement of self-antigen-derived peptides presented by major histocompatibility complex (MHC) antigens. The expression of thousands of tissue-specific self-antigens (TSAs) by medullary thymic epithelial cells (mTEC) is directly promoted by AIRE, a poorly understood transcriptional regulator (Mathis and Benoist, 2009; Klein et al., 2014). Reflecting its importance, AIRE deficiency is defined by the APS1/APECED syndrome for which autoimmune polyendocrinopathy and chronic mucocutaneous candidiasis are pathognomonic (Nagamine et al., 1997).

There are also several mechanisms of peripheral T cell tolerance, including requirements for co-stimulatory signals for the activation of naive T cells; the expression of molecular "brakes" (e.g., CTLA-4, PD-1) by activated T cells; and the suppression of effector T cells in *trans* by *FOXP3*-expressing T-regulatory (T-reg) cells. Reflecting its importance, *FOXP3* deficiency is defined by early-onset, life-threatening autoimmunity (Bennett et al., 2001; Wildin et al., 2001).

Central and peripheral tolerance mechanisms have likewise been hypothesized to shape the B cell compartment. Thus, self-reactive B cells developing in the bone marrow may be censored by clonal deletion, clonal anergy, or B cell receptor (BCR) editing in which secondary gene rearrangements replace the initial BCR with a new specificity (Goodnow et al., 2010; Pillai et al., 2011; Übelhart and Jumaa, 2015). Peripheral B cell tolerance is less well characterized, although some checkpoints have been inferred. For example, immature transitional B cells recently emigrated from the bone marrow contain many autoreactive and polyreactive cells, whereas there are relatively few among mature naive B cells, strongly suggesting that tolerance is imposed as transitional B cells differentiate into naive B cells (Wardemann et al., 2003).

Interestingly, this B cell checkpoint is T cell dependent, as reflected by its impairment in patients with T-reg deficiencies (Kinnunen et al., 2013). Likewise, CD40L and MHC class II deficiencies that each impair T-B interactions also display more autoreactive B cells (Meffre and Wardemann, 2008). These

 © 2016 The Authors. Published by Elsevier Inc.
This is an open access article under the CC BY license (http://creativecommons.org/licenses/by/4.0/).

A

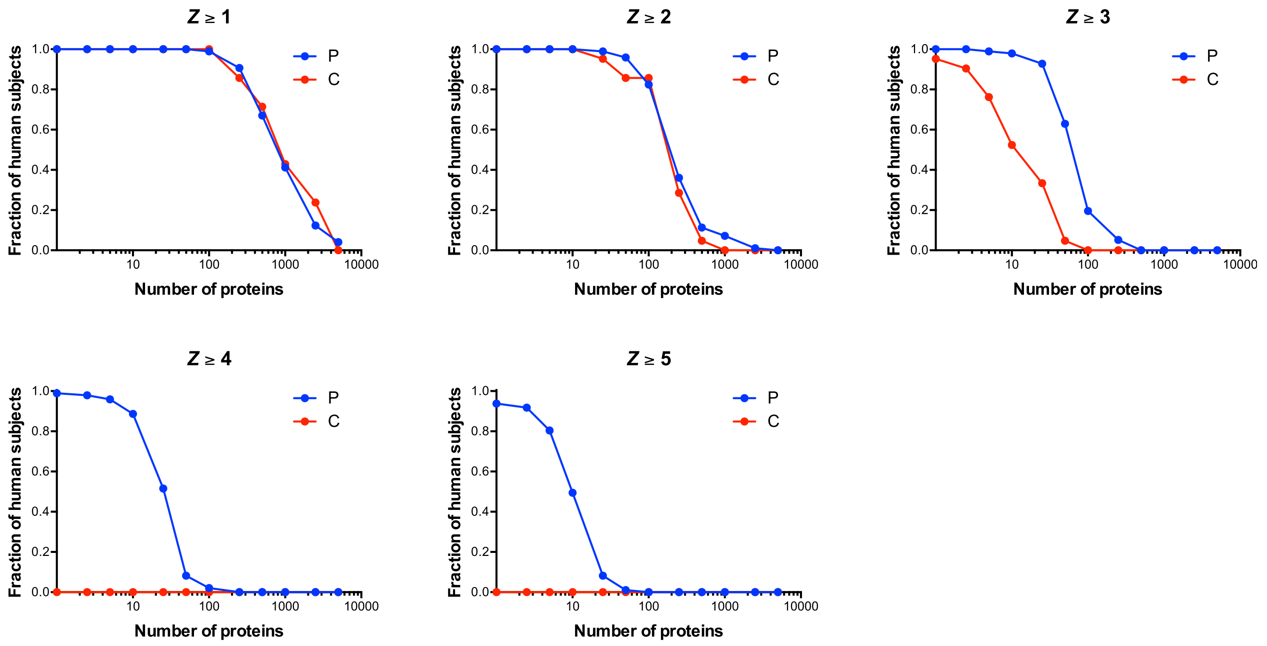

B

	Average # hits		# distinct proteins			Complexity factor	
	P	C	P	C	shared	P	C
Z ≥ 3	82.45	19.33	3731	406	126	0.47	1.00
Z ≥ 4	29.44	0.10	1536	2	0	0.54	0.95
Z ≥ 5	12.26	0.00	636	-	-	0.53	-

C D

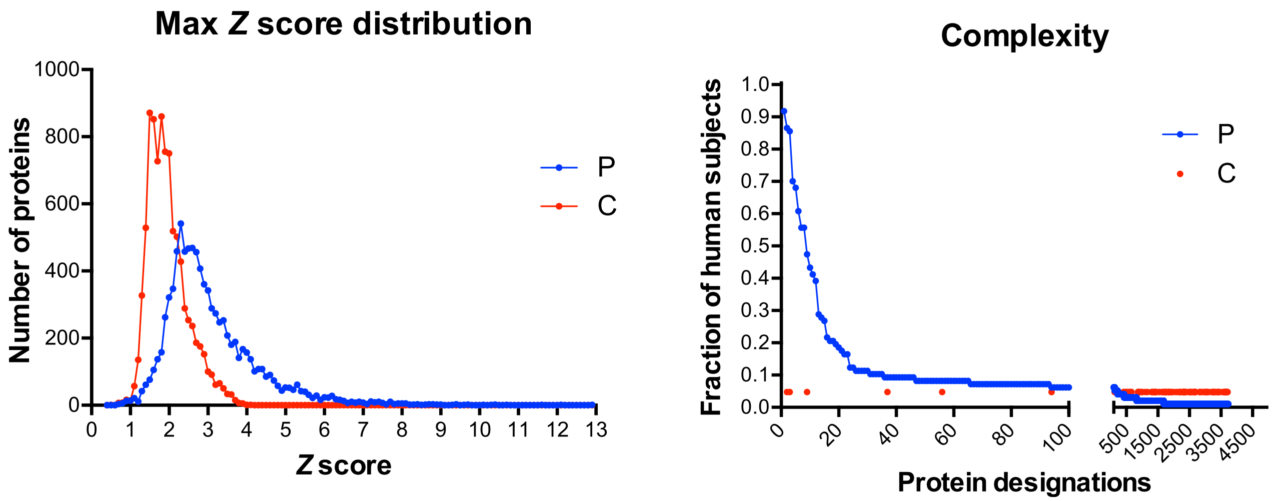

(legend on next page)

considerations raise the possibility that B cell tolerance is largely governed by the state of T cell tolerance.

Certainly, any autoreactive B cell that might progress through to the naive B cell compartment of a healthy individual should lack cognate autoreactive T cells to help it mature. Likewise, T cell help is required in the germinal center (GC) reaction in which B cells undergo somatic hyper-mutation (SHM) of the immunoglobulin (Ig) variable (V) region genes, thereby driving T cell-dependent selective expansion of clones with increased antigen affinity (Brink, 2014). The question that then arises is whether major defects in central T cell tolerance provoke wide-ranging losses of B cell tolerance at either or both of these stages.

An approach to assessing this is to examine B cell reactivities in AIRE-deficient APS1/APECED patients whose under-expression of TSAs in the thymus is predicted to lead to increased numbers of peripheral autoreactive T cells. Thus, there are reports of APS1/APECED patients carrying autoantibodies against twenty-five TSAs, with prevalence ranging from 6% to 69% (Kisand and Peterson, 2015). Their specificities include steroidogenic enzymes, consistent with the patients' polyendocrinopathies (Krohn et al., 1992; Uibo et al., 1994; Winqvist et al., 1993). In addition, most patients display autoreactivities toward type I IFNs and T helper (Th)-17-related cytokines, antibodies to which limit resistance to *Candida* infection (Kisand et al., 2010; Meager et al., 2006; Puel et al., 2010).

These findings notwithstanding, there has been no large-scale analysis of the scope and nature of autoantibodies in APS1/APECED patients, thereby resolving how T cell tolerance impacts upon B cell tolerance in humans. By analyzing 81 APS1/APECED patients, we found that each was much more likely than a healthy relative or an unrelated control to harbor strong serum reactivities toward ~100 human proteins. About 10 of those, including type I IFNs and interleukin-22 (IL22), were recognized by almost all patients, whereas others were mostly "private specificities." Hence, 81 patients collectively harbored antibodies toward >3,700 human proteins.

Focusing on antibodies to type I IFNs, IL22, and IL17, we found unexpectedly that most were reactive to conformational determinants and included highly mutated antibodies of subpicomolar affinity. Because their gemline counterparts were not self-reactive, B cell autoreactivity was most probably driven by self-reactive T cells in the GC reaction. The autoantibodies commonly neutralized their targets in vivo, and APS1/APECED patients with signature type 1 diabetes (T1D)-associated antibodies (e.g., anti-GAD65) commonly failed to develop T1D so long as they harbored powerfully neutralizing IFNα-specific antibodies. Thus, autoantibodies naturally arising in subjects with defective central T cell tolerance may be disease ameliorating.

RESULTS

High-Titer Autoreactivities in APS1/APECED

Sera from 81 APS1/APECED patients from discrete Finnish, Norwegian, Slovenian, and Sardinian cohorts were directed against a ProtoArray displaying ~9000 immobilized recombinant human proteins or protein fragments. Because some patients were sampled longitudinally, 97 sera were assayed in total. Control sera were from healthy first-degree relatives (n = 9) and healthy unrelated volunteers (n = 12) across the same age range. Data readouts for the binding of individual sera were normalized by applying robust linear modeling (Sboner et al., 2009), whereafter each signal was assigned a *Z* score denoting the number of standard deviations (SD) above or below the mean of the combined healthy relatives and controls.

Most patients and the combined controls displayed *Z* scores of 1–2 for ~200 proteins (Figure 1A). However, when the convention was employed of defining $Z \geq 3$ as bona fide positives, the patients segregated from the two control cohorts, considered either jointly or separately. Thus, each control serum displayed reactivities of $Z \geq 3$ toward an average of $\leq$20 proteins, with most recognizing < 10 (Figures 1A, 1B, and S1A). Given that there was inter-individual variation, the 21 control sera collectively displayed $Z \geq 3$ reactivities toward 406 distinct proteins, i.e., ~5% of those displayed on the array (Figure 1B). For only 2 proteins was $Z \geq 4$, and for none was $Z \geq 5$ (Figures 1A and 1B). Hence, as expected, the control cohorts largely lacked high-titer serum autoreactivities.

Conversely, most patients at any one time displayed $Z \geq 3$ autoreactivities toward $\geq$ 80 proteins (Figures 1A, 1B, and S1A). These data were re-analyzed with stringent procedures to minimize false-positives, including exclusion of any signals that might have arisen from cross-sample print contamination. With this achieved, the patients' "private" autoantibody repertoires collectively detected 3,731 distinct targets (Figure 1B). Furthermore, almost all patients displayed $Z \geq 4$ scores for at least 10 proteins (mean of ~30), collectively recognizing > 1,500 proteins, and > 50% of patients displayed $Z \geq 5$ scores for $\geq$ 10 proteins (mean of > 12), collectively recognizing 636 proteins (Figures 1A and 1B). Hence, high-level reactivity toward multiple self-proteins was a disease-defining property. This was further illustrated by the qualitative difference in *Z* score distribution curves for patients versus controls, which cannot simply be explained by there being 5-fold more patient sera (Figure 1C). Thus, whereas sampling greater numbers would likely have increased the protein species detected by control cohorts at $Z \geq 4$, it would not bridge the 1,000-fold gap between two proteins detected by 21 control sera versus > 1,500 proteins detected by 97 patient sera (Figure 1B).

Figure 1. Immune Response Profiling of APS1/APECED

(A) Distributions of hits between patients and controls at different *Z* scores.

(B) *Z* scores for all samples against all protein features and mean hits for each group calculated for $Z \geq 3$, $Z \geq 4$, and $Z \geq 5$. The number of distinct proteins targeted in each group (P, n = 97; C, n = 21) at *Z* scores denoted. The complexity factor was calculated by dividing the number of distinct proteins by average number of hits per patient.

(C) The max *Z* score distribution of all proteins in patient and control groups.

(D) Fraction of patients recognizing each of 3,731 proteins at $Z \geq 3$. Red dots depict 126 proteins shared between patients and controls.

In sum, 81 different patients collectively displayed strong reactivities to >40% of human proteins arrayed. For most proteins (blue dots 13–3731, Figure 1D), reactivities were spread across the cohort, reflecting high inter-patient variation, whereas ~12 proteins (blue dots 1–12, Figure 1D), including several type I IFNs, were recognized by > 60% of patients, as reported (Meager et al., 2006). However, the "public specificities" were not enriched among the 126 reactivities shared between patients and controls at $z > 3$ (red dots, Figure 1D), emphasizing that their common autoantigenicity is unique to the patients. Patient autoreactivity frequencies were largely comparable across geographical locations, albeit somewhat less in Norway and Slovenia, and age ranges (Figures S1B and S1C). Indeed, most anti-IFN autoantibodies of APECED patients were reported to increase early in life and remain stable thereafter (Meager et al., 2006; Wolff et al., 2013).

The collective targets of patient antibodies included intracellular, trans-membrane, and secreted proteins. Because many proteins displayed on the ProtoArray may be denatured, there may be false-negatives that underestimate patient reactivities to conformational determinants. Although a detailed analysis of the types of proteins targeted will be presented, it is evident that the proteins most commonly detected by patient sera included numerous cytokines, particularly type I IFNs, for which reason this study focuses on the nature of those autoreactivities.

Strong, Selective Anti-Cytokine Reactivities

Human type I IFN genes include 13 IFNα genes, 1 IFNβ gene, and 1 IFNω gene. There is also a type II IFNγ gene and three type III IFNλ genes. IFNγ is largely limited to lymphocytes, whereas type I and type III IFNs are broadly expressed, with their functional uniqueness and/or redundancy unresolved (Ivashkiv and Donlin, 2014). As assessed by ProtoArray, patient sera showed significantly stronger reactivities than controls toward all IFNα subtypes, albeit the reactivities to some (e.g., α1/13, α5, and α14) were higher than those to others (e.g., α2, α16, and α21) (Figure 2A). The differential between patients versus controls was emphasized by luciferase-based immunoprecipitation (LIPS) in which many target proteins were recognized in their native conformations (Figure 2B). Many patients showed strong reactivities to IFNω but rarely toward IFNβ (Figure 2B) and never toward IFNκ and IFNε, two phylogenetically distant type I IFNs (data not shown). By contrast, patient sera harbored reactivities significantly above controls toward IL1α, IL5, IL6, IL17A, IL17F, IL20, IL22, IL28A (IFNλ2), IL28B (IFNλ3), and IL29 (IFNλ1) (Figure 2C). Whereas reactivities toward some targets (e.g., IL17F, IL22) were common to most patients, reactivities toward others (e.g., IL20, IL28, IL6) were not (Table S1), and with the exception of IL5, patient sera mostly did not detect either Th2 cytokines (e.g., IL4 and IL13) or IL21, a Tfh (T follicular helper) cell cytokine that drives high-affinity antibody maturation. There were also no reactivities toward G-CSF and GM-CSF (Table S1), which drive the development of myeloid cells associated with the patients' inflammatory endocrinopathies.

Cytokine reactivities were largely validated by ELISA, which confirmed that IFNγ was only rarely and weakly recognized by patient sera (Figure 2D; Table S1) and that there was no reactivity toward TNFα (data not shown). By contrast, ELISA revealed autoantibodies toward IL32α and IL32γ, two poorly characterized proinflammatory cytokines (Figure 2D; Table S1). In sum, 81 APS1/APECED patient sera collectively displayed strong reactivities to a very selective subset of human cytokines.

Very High-Affinity Human Antibodies

To understand the nature of patient serum reactivities, nine IFNα-specific monoclonal antibodies (mAbs) were derived by limit-dilution cloning from memory B cells of four patients. Two were characterized in detail (26B9 and 19D11), whereas a more limited analysis of the others strongly argued that the properties of 26B9 and 19D11 were generally representative of patients' cytokine-specific antibodies. First, their V_H and $V\kappa$ sequences were highly mutated relative to their germline counterparts, with non-conservative replacements enriched in complementarity-determining regions (CDRs), as expected (white; Figure 3A). The antibodies bore no obvious resemblance to each other in V-gene segment or CDR3 usage. Conversely, a third anti-IFNα antibody, 50E11, shared with 19D11 the same V_H (IGHV1-69) and junctional (IGHJ4) segments and a very similar light chain (IGKV3-11 versus V3-20) (Figure S2A). However, there were very different template-independent nucleotide insertions in the V_H CDR3s of 19D11 and 50E11, and the somatic mutation patterns were different: whereas 19D11 and 26B9 showed high mutation frequencies in V_H CDR2 and $V\kappa$ CDR1, 50E11 did not (Figures 3A and S2A).

The recombinant antibodies 26B9 and 19D11 harvested from transfected CHO cells were immobilized on surface plasmon resonance (SPR) chips over which were run recombinant human IFNα2b, IFNα4, IFNα14, and IFNω, the latter being recognized by 26B9 but not by 19D11 (Figure 3B). These experiments revealed very slow off-rates reflecting extremely high affinities of the antibodies for their targets, ranging from $K_D = 3.28e^{-14}$M for 26B9 toward IFNα14 to $K_D = 2.09e^{-11}$M for 26B9 toward IFNα2b (Figures 3B and 3C). Sub-picomolar/near-femtomolar dissociation constants were likewise shown by 19D11 (Figures 3B and 3C). Thus, APS1/APECED patients harbor some of the strongest affinity antibodies described.

18-mer peptides spanning IFNα2b and IFNω were used to map linear epitopes recognized by 26B9 and 19D11. However, no specific reactivities were detected (data not shown), consistent with the antibodies binding conformational determinants shared by several type I IFNs. Also, the antibodies reacted poorly or not at all to mouse IFNs (Table S2).

To investigate the origins of the high-affinity, conformation-specific antibodies, germline counterparts for 19D11, 26B9, and 50E11, albeit with the same CDR3-VDJ sequences, were expressed and tested by LIPS against recombinant human IFNα2b, IFNα8, and IFNα14. There was no measurable interaction with any target (Figure 3D), although the antibodies' quality was evident from their comparable detection by anti-human IgG (Figure S2B). These data argue that the strong autoreactivity toward IFNs developed de novo during affinity maturation, rather than being an intrinsic property of the germline repertoire that is enhanced by affinity maturation.

The high affinities of 26B9 and 19D11 were not unique. Thus, a patient-derived IgGκ mAb (20A10) specific for IL20 (which is not a target detected by most patients; Figure 2C; Table S1)

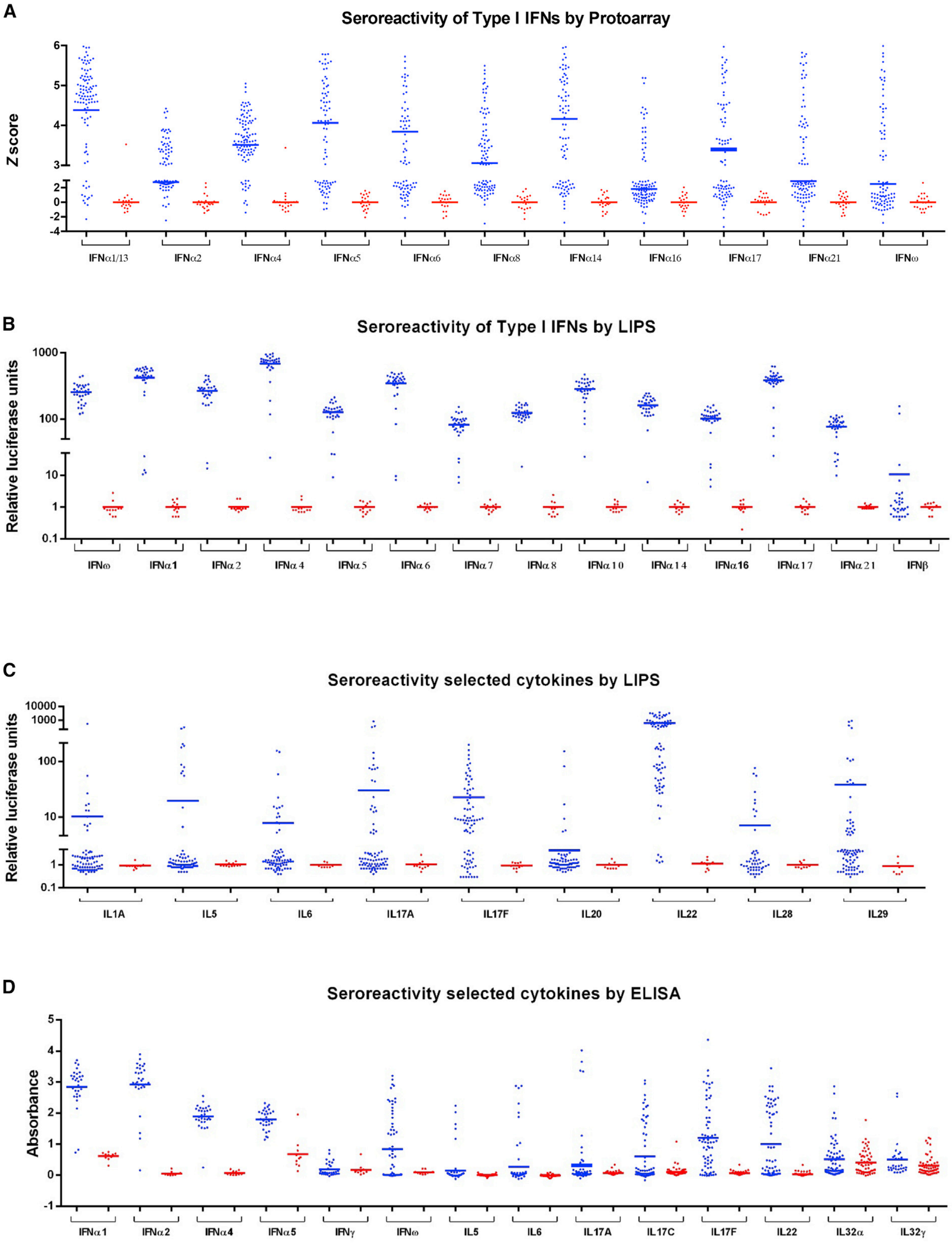

Figure 2. Serology of APS1/APECED to IFNs and Other Cytokines

Seroreactivity of APS1/APECED patients (blue) and contols (red) toward selected interferons and cytokines as measured in ProtoArray (A), LIPS (B and C), and ELISA (D).

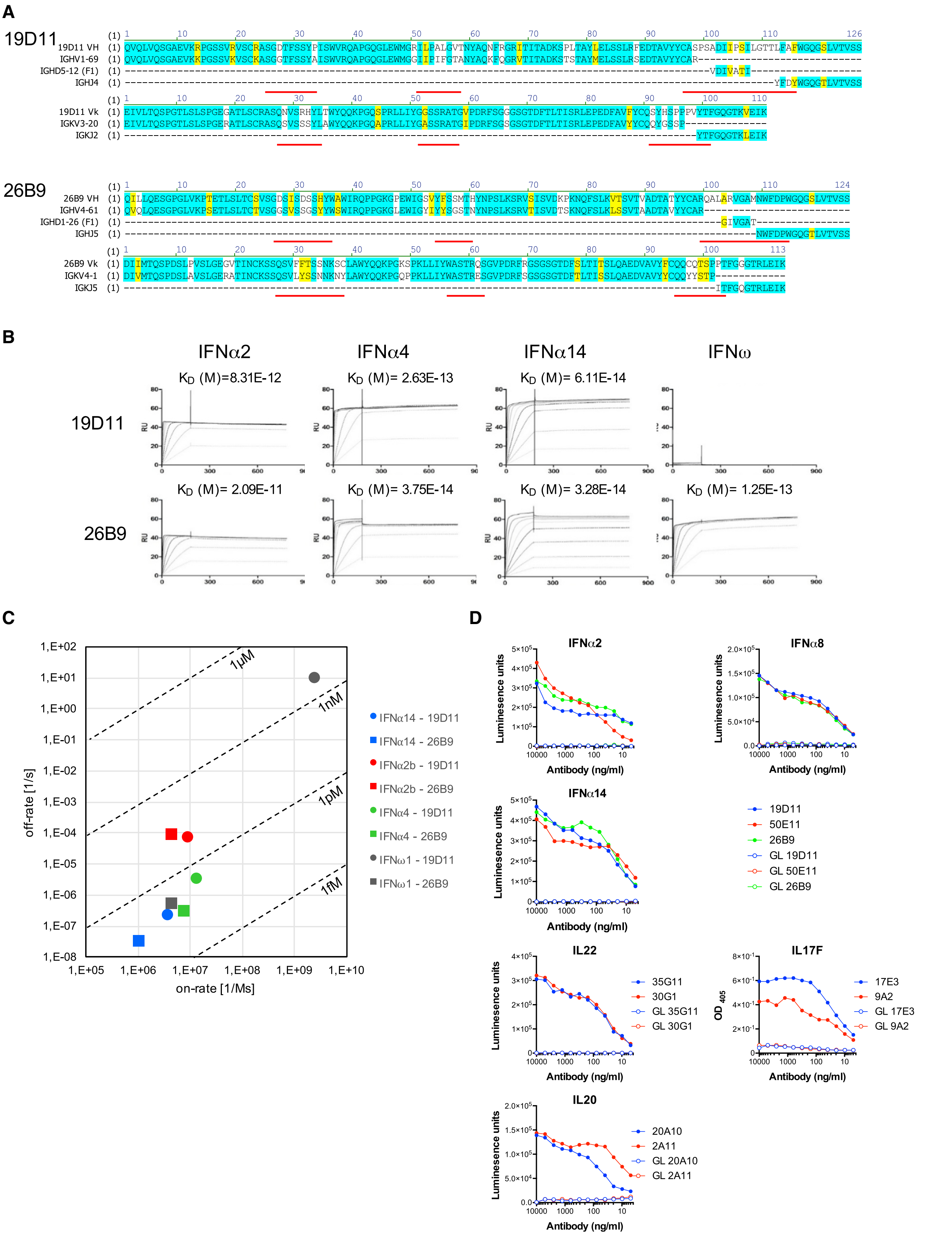

(legend on next page)

displayed a K_D of 9.1e^{-14}M, (Table S3). Likewise one IgGκ mAb (17E3) and one IgGλ mAb (24D3), each specific for IL17F, displayed dissociation constants of <10 pM, and one IgGκ antibody (30G1) and one IgGλ antibody (35G11) specific for IL22 displayed dissociation constants of 37 pM and 39 pM, respectively. As a comparison, a CHO cell-expressed form of fezakinumab, a humanized anti-IL22 mAb tested in the clinic, displayed a K_D of 54 pM (Table S3). The only exception to this pattern was 2C2, an IgGλ mAb specific for IL32γ (for which no human antibody has been reported), which displayed nanomolar dissociation (Table S3).

Similar to IFNα antibodies, most cytokine-specific antibodies did not detect linear peptides from relevant target proteins, strongly suggestive of complex conformational determinants (data not shown). The one exception was 20A10, which bound to an IL20 peptide and within which key amino acids were identified by mutagenesis (Figures S2C and S2D).

The antibody sequences of IL17F-reactive 17E3 and 9A2 and of IL22-reactive 30G1 and 35G11 displayed myriad non-conservative mutations enriched in the CDRs. Again their germline counterparts did not detect the respective targets (Figures 3D, S2E, and S2F). Moreover, neither patient-derived antibodies nor their germline counterparts showed any general autoreactivity (judged by immunofluorescent staining of tissue sections or HEp-2 cells) or reactivity to *Candida albicans*, thus arguing against candida infection being the trigger for autoantibody generation (data not shown).

The highly mutated CDRs of all studied antibodies suggested that they derived from GC reactions that partially rely on Tfh cells. Aberrant generation and/or activation of Tfh cells has been described in several autoimmune diseases (Ueno et al., 2015), but when four pediatric and four adult APS1/APECED were compared to controls, we found no differences in the percentages of circulating CXCR5$^+$ Tfh cells, or their activation state, as judged by ICOS (inducible costimulator) and CCR7 levels (Figure S3).

Biologically Active Human Antibodies

To test the biological activities of 19D11 and 26B9, HEK293 cells transfected with type I IFN-stimulated response elements (ISRE) fused to firefly luciferase were treated with recombinant forms of each of 12 IFNα subtypes and IFNω in the presence or absence of increasing concentrations of 19D11 or 26B9. Following treatment, firefly luciferase values were normalized to those of co-transfected *Renilla* luciferase, so as to control for variations in transfection efficiency. Both antibodies strongly inhibited the IFN-dependent response, with median IC_{50} values of 2.83 ng/ml for 26B9 and 0.9 ng/ml for 19D11 (Figure 4A; Table S4). By comparison, median IC_{50} values of 76.24 ng/ml and 10.86 ng/ml, respectively, were displayed by in-house-generated recombinant sifalimumab and rontalizumab, two anti-IFN mAbs used in clinical trials for systemic lupus erythematosus patients (Table S4).

Predictably, the antibodies varied in their inhibition of IFN-stimulated responses. Thus, 26B9 neutralized IFNω, but not IFNα16, and only poorly inhibited IFNα8 (Figure 4A; Table S4). Likewise, in the same assay, rontalizumab failed to efficiently neutralize IFNα6, IFNα7 and IFNα10, whereas sifalimumab neutralized several IFNα subtypes only weakly. By contrast, 19D11 neutralized all 12 IFNα subtypes tested (Table S4).

Patient-derived IFN-specific mAbs were also assessed for their capacity to inhibit STAT1 phosphorylation in cells treated with each of 12 IFNα subtypes, IFNω, IFNβ, or IFNγ (Figures 4B, 4C, and 4D). As predicted from the luciferase assay, 19D11 inhibited STAT1 phosphorylation levels (normalized to total STAT1 or tubulin) driven by all IFNα subtypes but did not affect responses to IFNω, IFNβ, or IFNγ. By contrast, 25C3, an additional patient-derived mAb (Table S2), was highly selective for discrete IFNα subtypes, whereas other antibodies tested, including 26B9, showed neutralization profiles between those of 19D11 and 25C3 (Figures 4B–4D). Only 26B9 and 31B4 neutralized IFNω, and none neutralized IFNβ or IFNγ. By comparison, sifalimumab, rontalizumab, and AGS-009 (another IFNα-targeting mAb in clinical development) showed variable and less uniform inhibition of STAT1 phosphorylation induced by different IFNα subtypes (Figure S4A).

The striking biological activities of patient mAbs were not limited to those specific for IFNs in that potent functional target neutralization was shown by mAbs targeting IL17F, IL22, IL32γ, and IL20, respectively (Figure S4B).

Biologically Active Human Antibodies In Vivo

We next asked whether patient autoantibodies could functionally neutralize targets in vivo. To test this, mice were treated intraperitoneally (i.p.) with a single aliquot of antibodies 26B9, 19D11, or sifalimumab, and their ears inoculated intradermally (i.d.) on days 1, 3, 6, and 8 with recombinant human IFNα5 or IFNα14 (Figure 5A) and IFNω (data not shown). Relative to repeated inoculation with vehicle/PBS, the cytokines induced ear swelling, reflecting an inflammatory response that includes rapid TNFα and IFNγ induction (Figures S5A and S5B). This ear swelling was significantly inhibited by single injections of antibodies (Figure 5B). Again, neutralization varied toward the effector IFNα subtype: 26B9 and 19D11, but not sifalimumab, largely ablated the IFNα5 response, whereas all three partially yet significantly limited swelling induced by IFNα14 (Figure 5B).

Specific, antibody-mediated neutralization in vivo was likewise seen when the same assay was applied to human IL17F

Figure 3. Affinity of Patient-Derived mAbs

(A) Amino acid sequences of 26B9 and 19D11 anti-IFN antibodies aligned with closest corresponding germline IgV_H, D_H, J_H, V_L, and J_L sequences. Identities highlighted in blue; conservative mutations in yellow; non-conservative in white; CDRs underlined in red.

(B) Plasmon resonance data: antibodies 19D11 and 26B9 were immobilized on Biacore chips; different concentrations of recombinant human IFNα2b, IFNα4, IFNα14, and IFNω were passed over; response units were recorded; and dissociation constants (K_D) calculated.

(C) Scatter chart of K_D values derived from (B).

(D) Binding determined by LIPS of APS1/APECED-derived mAbs and of germline counterparts to IFNα2, IFNα8, IFNα14 (19D11, 50E11, and 26B9), IL22 (35G11 and 30G1), and IL20 (20A10 and 2A11). Binding to immobilized IL17F (17E3 and 9A2) was determined by ELISA.

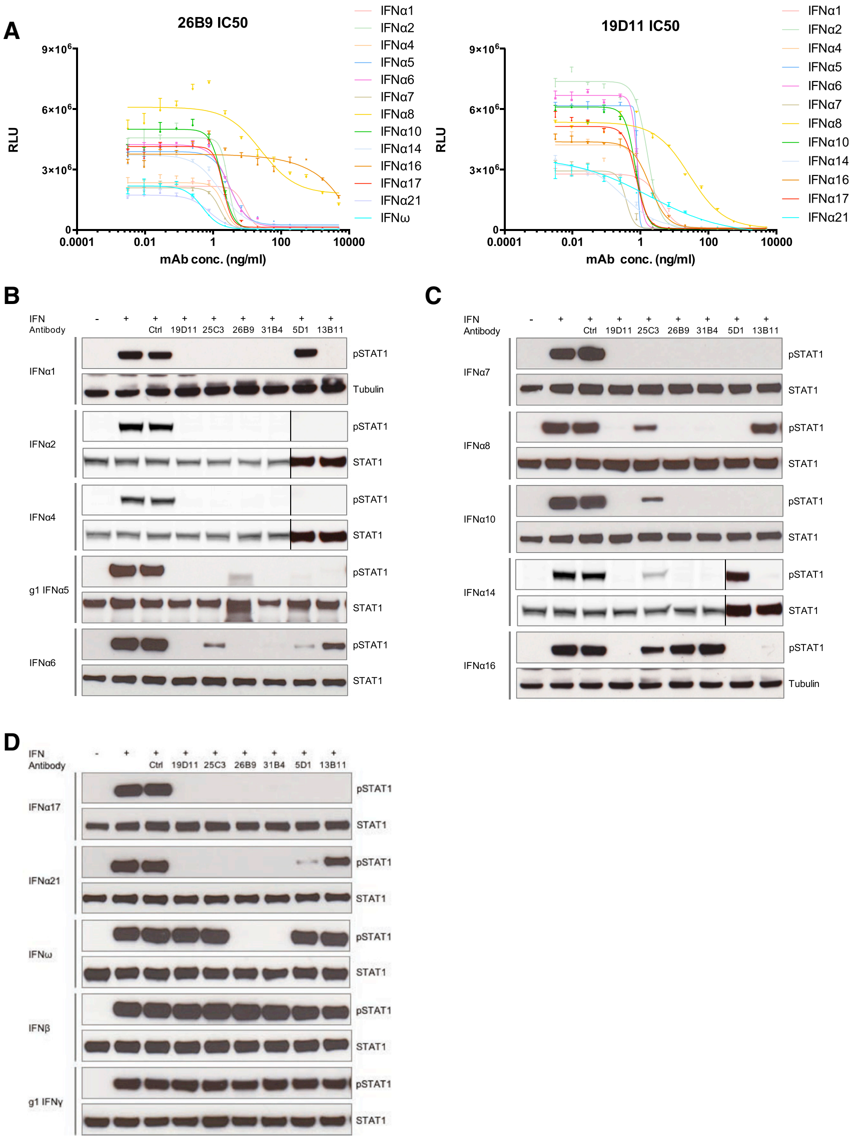

Figure 4. In Vitro Neutralization

(A) IC_{50} analysis of APS1/APECED-derived anti-IFN mAbs 19D11 and 26B9 in HEK293T MSR cells transfected with ISRE dual-luciferase reporter constructs and treated with IFNα subtypes shown. Error bars correspond to SEM of multiple measurements.

(B–D) IFN-induced STAT1 tyrosine phosphorylation detected by western blot and normalized to total STAT1 or to tubulin levels as loading controls. Vertical lines in (B) and (C) denote cropped lanes.

or IL32γ (Figures 6A and 6B). For IL17 neutralization, the data are clearly consistent with the known capacity of APS1/APECED patients' antibodies to neutralize Th17-family cytokines (Kisand et al., 2010; Puel et al., 2010), thereby predisposing to *Candida* infection.

Additionally, the detection of mouse IL22 by antibody 30G1 offered an opportunity to measure its bio-activity toward endogenous IL22, a primary effector of imiquimod (IMQ)-induced dermatitis used to model psoriasis (van der Fits et al., 2009). IMQ-induced pathology measured by modified PASI scoring was significantly ameliorated by 30G1 relative to IgG control, particularly following an initial inflammatory response (Figures 6C and S6). Again, 30G1 was at least as effective as an in-house-expressed anti-IL22 antibody, fezakinumab (see above) (Figure 6C). Collectively these data establish the capacity of patient anti-cytokine antibodies to limit pathologies induced by their targets in vivo.

Clinical Correlates of Neutralizing Antibodies

Given the results from animal models, it was appropriate to consider the potential impact of APS1/APECED antibodies in the patients themselves. Because circulating IFNα levels are extremely low in human peripheral blood, even following vaccination (Sobolev et al., 2016), circulating IFN levels do not offer robust biomarkers of anti-IFNα antibodies. Neither does measurement of interferon-stimulated genes (ISGs) because many, e.g., CXCL10, can be upregulated by type II IFNs (Welcher et al., 2015). By contrast, antibody activities may be reliably reflected in discrete pathologies, as in the correlation of anti-IL22 with candidiasis.

In this regard, many datasets, particularly in mouse models, suggest that type I IFN contributes to type 1 diabetes (T1D) (Carrero et al., 2013; Downes et al., 2010; Foulis et al., 1987; Huang et al., 1995; Li et al., 2008). Although APECED/APS1 patients by definition suffer from polyendocrinopathy, T1D affects only ~10%–20% of patients and manifests primarily in adulthood (Husebye et al., 2009; Kisand and Peterson, 2015). This is despite the fact that radioimmunoassays have revealed that many APS1/APECED patients carry GAD65-reactive autoantibodies, a clinically applied biomarker for likely onset of T1D (Ziegler et al., 2013). Consistent with this, ProtoArray and LIPS data showed that many patients carried GAD65- and/or GAD67-reactive antibodies, but among them relatively few presented with T1D (red dots, Figures 7A and 7B). Collectively, these many observations suggest that patients at risk of T1D, as judged by anti-GAD65/GAD67, might fail to develop T1D if they harbored powerfully neutralizing anti-IFNα antibodies. Indeed, we reported a seemingly exceptional APS1/APECED patient, completely lacking IFNα-neutralizing antibodies and presenting with T1D (Kisand et al., 2008).

To investigate this, the 8 patients presenting with T1D (red dots, Figure 7B; mean age ± SD, 48 ± 11 years) were compared with an available cohort of 13 patients without

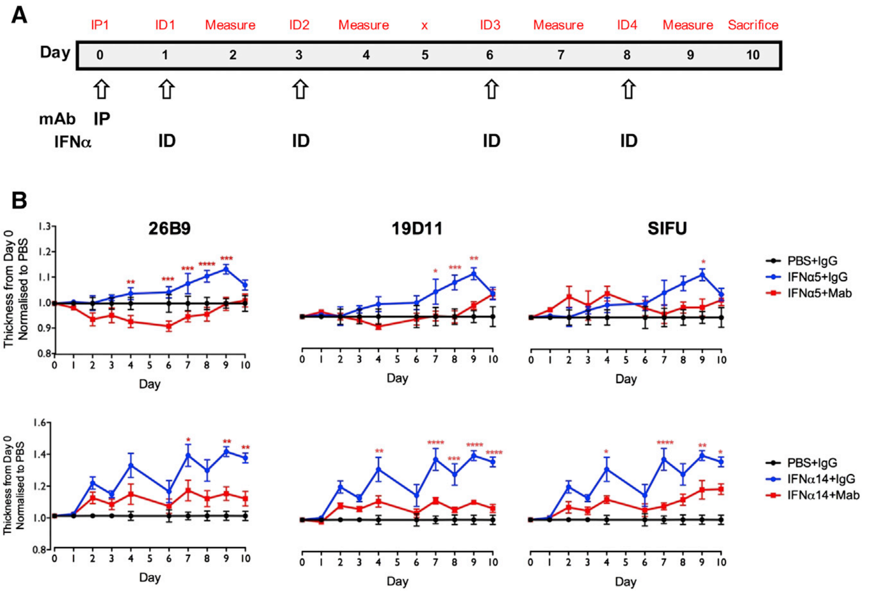

Figure 5. Biological Activity of IFN mAbs

(A) Experimental timeline: mAb administered i.p. at day 0; human IFNα administered i.d. on days 1, 3, 6, and 8. Ear thickness measured on all days (prior to cytokine injection) except for day 5.

(B) I.p.-administered IFN mAbs reduced IFNα-induced ear inflammation.

Significance calculated by two-way ANOVA, with *p $\leq$ 0.05, **p $\leq$ 0.01, ***p $\leq$ 0.001, and ****p $\leq$ 0.0001. Error bars denote SEM.

T1D but with strong GAD65 reactivity (relative luciferase units > 5) (blue dots, Figure 7B; mean age ± SD, 31 ± 12 years). Consistent with T1D developing in adult APS1/APECED patients, GAD65 reactivities mostly arose post-adolescence, and hence the patient cohorts comprised 20 adults and one 8 year old.

As expected, all 21 patients harbored antibodies to IFNα and IFNω (see Figure 2B), but when tested for IFNα and IFNω neutralization, the antibodies showed a striking segregation with clinical status (Figures 7C, 7D, and S7A): patients without T1D collectively neutralized all IFNα subtypes, whereas those with T1D showed only low or negligible neutralization. Particularly strong differences were seen vis-a-vis IFNα1, IFNα2, IFNα5, IFNα8, IFNα14, and IFNα17 neutralization (Figure 7C). The two subgroups of the 21 patients also showed statistically significant differences in neutralizing IFNω, but the difference was weaker than for IFNα (Figure S7A). Interestingly, the two GAD65-reactive non-diabetics who displayed relatively low IFNα neutralization were young adults who may be en route to developing T1D.

In a small subcohort of GAD65/67-reactive patients for whom longitudinal samples were available, the three T1D patients (red bars) again showed lower IFNα neutralization relative to the two patients without T1D. Moreover, one patient was able to neutralize IFNα4 in 1978 but by 2012 could no longer do so and presented with T1D (Figure S7B).

Such striking correlations with T1D (Figure 7D) were not evident for any other naturally arising anti-cytokine antibodies, supporting the view that IFNα may contribute critically to the natural progression of T1D. Moreover, although the data do not prove that active anti-IFN antibodies underpin selective protection from T1D, they provide a firm foundation for exploring the potentials of APS1/APECED-derived autoantibodies to ameliorate other major diseases that are rarely if ever present in APS1/APECED patients.

DISCUSSION

This analysis of the impact of AIRE deficiency on human B cells has revealed a signature pattern of humoral autoreactivity with general implications for our understanding of autoimmunity. First, the autoantibodies studied were mostly extremely high affinity and specific for native conformational epitopes. These properties were shared by antibodies specific for cytokines targeted by most patients (e.g., IFNα, IL17, IL22) and by antibodies specific for IL20 to which few patients displayed reactivity. Because such properties are very rare among antibodies raised by immunization, when B cells are primed de novo to antigen for short periods of time, it seems inappropriate to continue to model one type of mAb on the other.

Second, essentially all 81 APS1/APECED patients studied showed strong reactivities toward a common set of 10–15 proteins, coupled with patient-specific reactivity profiles toward 80–90 additional proteins. This limited frequency (< 1% of proteins displayed on the array) is consistent with a recent report that B cell tolerance was not globally disrupted in 51 APS1/APECED patients sampled (Landegren et al., 2016). Nonetheless, the patient-to-patient variation in reactivity profiles meant that the 97 sera analyzed in our study collectively harbored antibodies toward over 3,500 proteins.

The patient-to-patient variation argues that B cell autoimmunity resulting from AIRE deficiency is not simply an amplification of sporadic, low-level autoreactivities seen in healthy controls but has distinct origins. By this perspective, defects in central T cell tolerance may underpin other autoimmune and autoinflammatory pathologies attributed to high-affinity autoantibodies. Whereas this contrasts with the widely held view that autoimmune diseases mostly reflect peripheral tolerance defects, it aligns with data that central tolerance defects contribute to the NOD mouse model of T1D (Geng et al., 1998; Zucchelli et al., 2005). Moreover, wherever autoantibodies reflect central T cell tolerance defects, donor-to-donor variation is to be expected, as individuals will generate distinct TCR repertoires via quasi-random gene rearrangements, will be exposed to different physiologic and environmental triggers that promote the selective outgrowth of autoreactive T cell clones, and will differ in immune response modifier genes (e.g., HLA) that regulate the magnitude of antigen-specific responses.

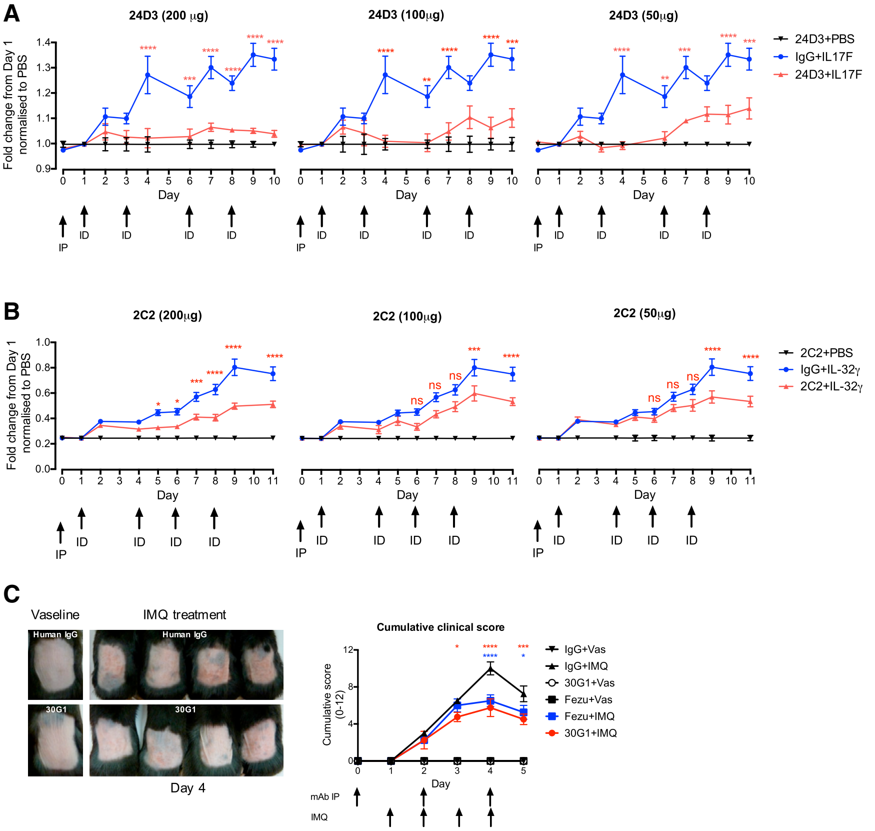

Figure 6. In Vivo Activity of Cytokine-Reactive mAbs

(A) mAb administered i.p. at day 0, and human IL17F administered i.d. on days 1, 3, 6, and 8. Ear thickness measured on all days (prior to cytokine injection) except day 5.

(B) As in (A), but with human IL32γ administered i.d.

(C) anti-IL22-specific mAb injected i.p. into 9-week mice prior to and during IMQ treatment. Efficacy measured by Psoriasis Area and Severity Index (PASI).

Significance calculated by two-way ANOVA, with $^*p \leq 0.05$, $^{**}p \leq 0.01$, $^{***}p \leq 0.001$, and $^{****}p \leq 0.0001$. Error bars denote SEM.

Autoantibodies to some non-tissue-restricted antigens, including multiple type I IFNα subtypes, are displayed by almost all patients, sometimes early post-partum (Wolff et al., 2013). Most likely, the immunogenicity of these proteins arises by mechanisms distinct from those shaping patient-specific autoantibody repertoires. Possibly the public autoantibodies arise from a direct impact of AIRE deficiency on B cell tolerance, for example, via the dysregulation of AIRE-expressing thymic B cells that resemble GC B cells by several criteria (Yamano et al., 2015). Arguing against this, however, autoantibodies to type I IFNs, Th17 cytokines, and additional self-proteins are found in thymoma patients with AIRE-sufficient B cells (Kisand et al., 2011; Meager et al., 1997; Wolff et al., 2014). This likewise argues against autoantibodies to type I IFNs and Th17 cytokines originating from defects in lymph node AIRE$^+$ cells termed eTACs (Gardner et al., 2008). Although studies in mice have suggested tolerizing roles of eTACs, the functions of their rare human counterparts are unknown (Poliani et al., 2010).

AIRE deficiency may, however, act indirectly on thymic B cells, for example by hyperactivity of functionally competent thymic γδ cells (Ribot et al., 2009) that may likewise be dysregulated in thymoma. Such cells may create an intra-thymic milieu favoring priming rather than tolerance of thymic B cells toward proteins highly expressed in the thymus (Dudakov et al., 2012; Meager et al., 2006).

Notwithstanding this possibility, our findings suggest that high-affinity autoantibodies in APS1/APECED patients probably reflect dysregulated GC reactions, wherein autoreactive T cells, e.g., Tfh cells, that were not tolerized in the thymus promote the competitive outgrowth and affinity maturation of B cells that were initially primed to exogenous antigen(s) but whose mutated IgGs bind to self-proteins. Consistent with this, autoantibodies targeting thyroid-stimulating hormone receptor in Graves' disease cross-react to *Yersinia enterocolitica* antigens (Brink, 2014; Hargreaves et al., 2013), and activated peripheral blood Tfh cells correlate positively with serum autoantibodies and disease activity/severity in multiple autoimmune diseases (Ueno et al., 2015). Although our analysis of four adult and four pediatric APS1/APECED patients revealed no alterations in Tfh cell numbers relative to age-matched healthy controls, this did not exclude Tfh cells being enriched in autoreactive specificities. Moreover, no patients displayed neutralizing autoantibodies to IL21, a major mediator of Tfh cells in the GC.

This etiology of APS1/APECED B cell autoimmunity is strikingly similar to proposed origins of highly mutated anti-desmoglein-3 antibodies in autoimmune pemphigus (Di Zenzo et al., 2012) and of anti-GM-CSF antibodies pathognomonic in pulmonary alveolar proteinosis (Piccoli et al., 2015). In those studies, as in this, the closest germline counterparts ("unmutated common ancestors" [UCAs]) showed no reactivity toward the targets of the affinity-matured autoantibodies. By contrast, germline versions of antiviral antibodies showed only slightly reduced binding to target viral antigens (Corti et al., 2011, 2013). Moreover, it is not the case that UCAs intrinsically lack autoreactivity, as germline counterparts of some autoantibodies with few replacement mutations showed autoantigen reactivity in pemphigus patients (Cho et al., 2014). The underlying defect(s) in T cell tolerance that dysregulate affinity maturation in pemphigus, pulmonary alveolar proteinosis, and other organ-specific autoimmune diseases may be limited to few antigens, by contrast to broad-spectrum defects in APS1/APECED.

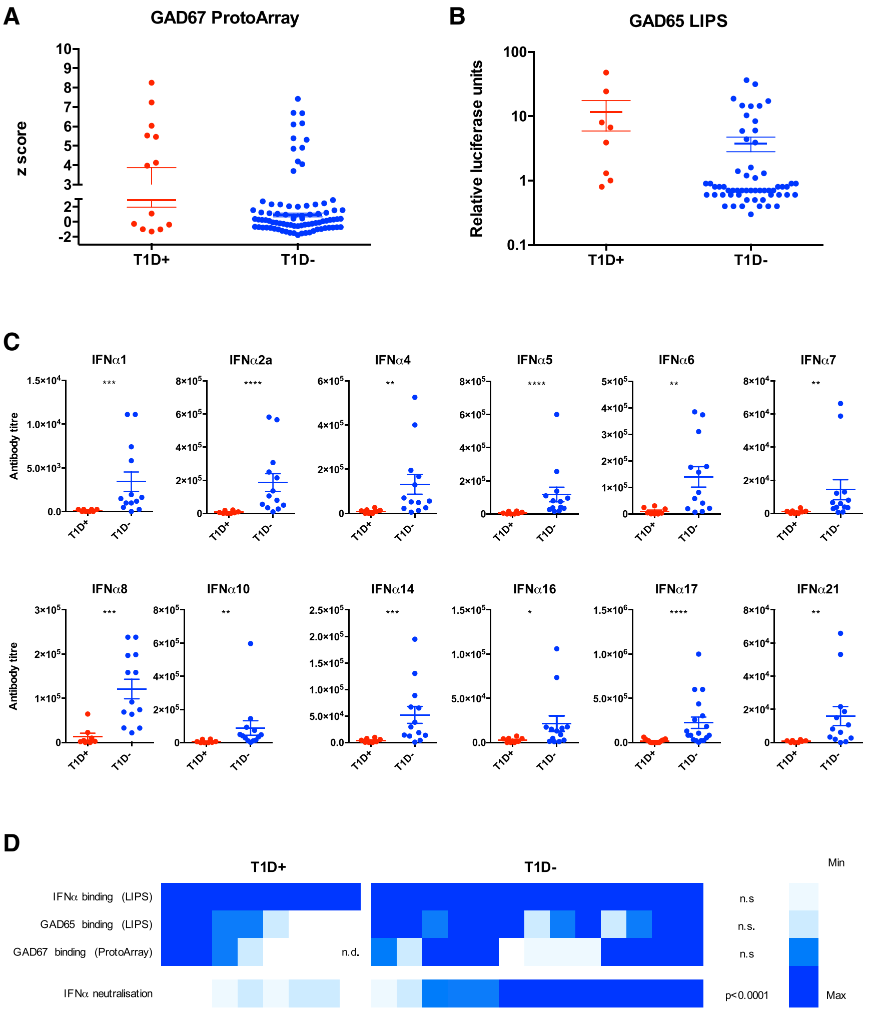

Figure 7. Clinical Correlation of T1D and IFN Neutralization

(A and B) Seroreactivity to GAD67 and GAD65 measured by ProtoArray and LIPS in APS1/APECED patients with (red) or without (blue) T1D.

(C) IFNα-neutralizing titers in patients with T1D (n = 8) and anti-GAD65 seropositive patients without T1D (n = 13). y axis shows inhibitory concentration IC_{50} reflecting serum dilutions at which IFN activity was reduced 50%.

(D) Heatmap of seroreactivity toward GAD67, GAD65, and IFNα analyzed by ProtoArray and LIPS combined with neutralization capacity in patients with and without T1D.

Significance calculated by Mann Whitney using GraphPad Prism v.6, with $^*p < 0.05$, $^{**}p < 0.01$, $^{***}p < 0.001$, $^{****}p < 0.0001$. Error bars denote SEM. Significance values in (D) compare $T1D^+$ and $T1D^-$ groups for each parameter.

That almost all APS1/APECED-derived mAbs were biologically active in vivo against a range of cytokine targets has profound implications for patients. Clearly, immune-effector responses may be reduced, as in the association of anti-IL22 with susceptibility to *Candidiasis* (Kisand et al., 2010). Likewise, gut barrier integrity may be compromised, leading to increased levels of anti-commensal antibodies (Hetemäki et al., 2016). Conversely, despite the common neutralization of IFNα and IFNω, APS1/APECED patients do not show severe viral infections, as were recently reported for a child genetically impaired in type I IFN (Ciancanelli et al., 2015). Possibly preserved IFNβ function mediates anti-viral protection in APS1/APECED patients.

On the other hand, some autoantibodies may target key mediators of immunopathologies, thereby ameliorating disease. Thus, a unique correlation was observed between antibody-mediated neutralization of IFNα and failure to develop T1D, providing a novel strand of support for animal studies arguing that targeting type I IFNs could be effective in T1D. The concept that naturally arising autoantibodies may be beneficial is not widely considered, despite its underpinning the widespread use of therapeutic mAbs. In this regard, it is striking that despite their severe flaws in central T cell tolerance, APS1/APECED patients do not present with systemic sclerosis, Sjögren's syndrome, MS, or SLE. These pathologies are considered to involve interplays of IL17/Th17 and type I IFNs—two main targets of APS1/APECED autoantibodies (Ambrosi et al., 2012). Likewise, Th17-driven psoriasis was diagnosed in only two of our patients, each of whom lacked autoantibodies to IL17A, IL17F, and IL22 (our unpublished data). Furthermore, atopy/allergy is seemingly rare among APS1/APECED patients, although whether anti-IL5 antibodies underpin this requires more study. For now, the data presented by this study strongly suggest that antibodies recovered from APS1/APECED patients include ones with profound therapeutic and diagnostic potential.

EXPERIMENTAL PROCEDURES

More details are available in the Supplemental Experimental Procedures.

Human Samples

Eighty-one APS1/APECED patients were diagnosed by mutational analysis of *AIRE* and by autoantibodies to type I IFNs. All provided informed consent, and many were analyzed previously (Kisand et al., 2011; Kluger et al., 2015; Meloni et al., 2012; Wolff et al., 2007). Approvals by local ethics committees are described in the Supplemental Experimental Procedures. Ages at serum sampling were 4–73 years; mean = 31.9. For protoarray there were 12 age-matched controls and 9 healthy first-degree relatives, and there were additional healthy controls for LIPS and ELISA.

Immune Response Profiling by ProtoArray

Sera of patients, healthy relatives, and controls were tested against > 9,000 human proteins displayed on the Human Protein Microarray v5.1 (ThermoFisher Scientific). Preprocessing methods were applied to account for technical variability. First, corresponding local background intensity was subtracted,

whereafter values were log-transformed and subjected to robust linear normalization (Sboner et al., 2009). Z scores were calculated as the number of standard deviations of the signal from the mean of the corresponding controls and healthy relatives; $Z \geq 3$ was considered positive. After scoring, stringent quality assessment was undertaken, including high correlation coefficients of duplicate spots of printed proteins (average r = 0.92), reactivity toward known autoantibody targets, and perfect correlation of signals for proteins spotted in different locations. Printing contaminants were identified as proteins showing high correlation coefficients with known APECED antibody targets and were further verified by cross-reference to another protoarray (5.0) used for 23 patients and 7 controls. Thus, 31 suspect false-positives were identified and excluded from further consideration.

Antibody Isolation and Cloning

Cloning, production, and purification of human mAbs were performed as described (patent application WO2013/098419). In brief, memory B cells ($CD22^+$, IgD^-, IgM^-, $CD3^-$, $CD8^-$, and $CD54^-$) were flow-sorted (MoFlo) from patient PBMC, incubated transiently with EBV-containing B95-8 supernatant (SN) for 3.5 hr at 37°C, and then incubated in Transferrin- and CpG-supplemented IMDM at 37°C, 5% CO_2, at 10 cells/well in 96-well plates coated with irradiated PBMC feeders. Short-term, oligoclonal B cell culture SN were analyzed for IgG and antigen-specific antibodies detected by ELISA and/or LIPS. Positive wells were harvested, cells single-cell-sorted into reverse transcriptase (RT) buffer (Life Technologies), and RT-PCR performed using Superscript III (Life Technologies) and random hexamers. IgG V_H, $V\lambda$, and $V\kappa$ regions were amplified from cDNA by two-step nested PCR reaction using Advantage 2 cDNA polymerase (Clontech) and primer mixes specific for germline families (VBASE database). Nested primers attached restriction sites for V-region cloning into expression vectors providing IgG1, Ig-κ, or Ig-λ constant regions. Recombinant antibodies were produced in HEK293T cells and antigen specificity analyzed by ELISA. Corresponding closest germline region sequences were identified using the VBASE2 database (Retter et al., 2005). CDRs were identified by IMGT definitions (Lefranc, 2003).

Complete Ig-V_H and V_L regions described in US7741449 (Sifalimumab), US7087726 B2 (Rontalizumab), US8361463 (ACO-1), and US20070258982 A1 (Fezakinumab) were ordered as CHO-codon-optimized synthetic constructs (GenScript) and expressed as above.

mAb Characterization In Vitro

EC_{50} binding of mAbs was determined by ELISA. Neutralizing capacities of type I IFN-specific mAbs were studied using phospho-STAT1 quantification in immunoblot and ISRE-luciferase reporter assay. IL17F, IL22, IL20, and IL32 neutralization assays were performed on respective responsive cell lines. mAB affinities were measured with a Biacore T200 (GE Healthcare). Epitope mapping used overlapping 18-mer peptides.

mAb Characterization In Vivo

C57BL/6J (WT; from Charles River) mice were administered i.p. with mAbs (day 0) and inoculated i.d. on days 1, 3, 6, and 8 with cognate human cytokines, IFNα2a, IFNα2b, IFNα4, IFNα14, IL17F, and IL32γ, and their ear thicknesses measured with a micrometer. For IL22 mAbs cross-reactive to mouse, bioactivity was assessed in imiquimod-treated mice.

SUPPLEMENTAL INFORMATION

Supplemental Information includes Supplemental Experimental Procedures, seven figures, and four tables and can be found with this article online at http://dx.doi.org/10.1016/j.cell.2016.06.024.

CONSORTIA

The members of the APECED patient collaborative are Antonella Meloni, Nicolas Kluger, Eystein S. Husebye, Katarina Trebusak Podkrajsek, Tadej Battelino, Nina Bratanic, and Aleksandr Peet.

AUTHOR CONTRIBUTIONS

S.M., A.M., and S.C.O. cloned monoclonal antibodies from patient samples, and K.J. and K.H. assisted. S.M., P.V., and A.M. characterized antibodies in vitro; M.W. and Y.H. did so in vivo. C.H. analyzed ProtoArray data and wrote and edited the paper. J.K. assayed neutralization by sera and Tfh subsets and performed LIPS. D.F. and H.P. analyzed ProtoArray data. K.M. and R.U. screened sera for T1D autoantibodies and tested germline antibody specificities. K. Krohn and A.R. developed the clinical database, sampled Finnish patients, and employed ELISA. A.S.B.W. sampled Norwegian patients, contributed to the clinical database, and assayed antibodies. APECED patient collaborative contributed to the clinical database and sampled respective patients. P.P., K. Kisand, and A.H. supervised research, reviewed data, and wrote and edited the paper.

ACKNOWLEDGMENTS

We are indebted to patients; to the Finnish APECED and Addison patients' association; and to attending physicians and carers. We thank M. Rothe, P. Adler, A. Remm, M. Pihlap, M. Karlsberg, A. Tallqvist, M. Tuukkanen, L. Prassmayer, M. Wördehoff, A. Peters, R. Repke, B. Mathis, and particularly E. Stuart and K. Henco for critical insight and support. We thank staff of the Biological Services Unit at King's College London. Funding was by the following: ImmunoQure AG, the Wellcome Trust, and CRUK (to A.H.) and Estonian Research Council grant IUT2-2 and European Union Project 2014-2020.4.01.15-0012 (to J.K., P.P., and K. Kisand). P.P., K. Krohn, K. Kisand, A.R., and A.H. are cofounders and shareholders of ImmunoQure AG, and A.M., C.H., P.V., S.C.O., and S.M. were/are employees of ImmunoQure AG.

Received: January 27, 2016
Revised: April 24, 2016
Accepted: June 10, 2016
Published: July 14 2016

REFERENCES

Ambrosi, A., Espinosa, A., and Wahren-Herlenius, M. (2012). IL-17: a new actor in IFN-driven systemic autoimmune diseases. Eur. J. Immunol. *42*, 2274–2284.

Bennett, C.L., Christie, J., Ramsdell, F., Brunkow, M.E., Ferguson, P.J., Whitesell, L., Kelly, T.E., Saulsbury, F.T., Chance, P.F., and Ochs, H.D. (2001). The immune dysregulation, polyendocrinopathy, enteropathy, X-linked syndrome (IPEX) is caused by mutations of FOXP3. Nat. Genet. *27*, 20–21.

Brink, R. (2014). The imperfect control of self-reactive germinal center B cells. Curr. Opin. Immunol. *28*, 97–101.

Carrero, J.A., Calderon, B., Towfic, F., Artyomov, M.N., and Unanue, E.R. (2013). Defining the transcriptional and cellular landscape of type 1 diabetes in the NOD mouse. PLoS ONE *8*, e59701.

Cho, M.J., Lo, A.S., Mao, X., Nagler, A.R., Ellebrecht, C.T., Mukherjee, E.M., Hammers, C.M., Choi, E.J., Sharma, P.M., Uduman, M., et al. (2014). Shared VH1-46 gene usage by pemphigus vulgaris autoantibodies indicates common humoral immune responses among patients. Nat. Commun. *5*, 4167.

Ciancanelli, M.J., Huang, S.X., Luthra, P., Garner, H., Itan, Y., Volpi, S., Lafaille, F.G., Trouillet, C., Schmolke, M., Albrecht, R.A., et al. (2015). Infectious disease. Life-threatening influenza and impaired interferon amplification in human IRF7 deficiency. Science *348*, 448–453.

Corti, D., Voss, J., Gamblin, S.J., Codoni, G., Macagno, A., Jarrossay, D., Vachieri, S.G., Pinna, D., Minola, A., Vanzetta, F., et al. (2011). A neutralizing antibody selected from plasma cells that binds to group 1 and group 2 influenza A hemagglutinins. Science *333*, 850–856.

Corti, D., Bianchi, S., Vanzetta, F., Minola, A., Perez, L., Agatic, G., Guarino, B., Silacci, C., Marcandalli, J., Marsland, B.J., et al. (2013). Cross-neutralization of four paramyxoviruses by a human monoclonal antibody. Nature *501*, 439–443.

Di Zenzo, G., Di Lullo, G., Corti, D., Calabresi, V., Sinistro, A., Vanzetta, F., Didona, B., Cianchini, G., Hertl, M., Eming, R., et al. (2012). Pemphigus

autoantibodies generated through somatic mutations target the desmoglein-3 cis-interface. J. Clin. Invest. *122*, 3781–3790.

Downes, K., Pekalski, M., Angus, K.L., Hardy, M., Nutland, S., Smyth, D.J., Walker, N.M., Wallace, C., and Todd, J.A. (2010). Reduced expression of IFIH1 is protective for type 1 diabetes. PLoS ONE *5*, e12646.

Dudakov, J.A., Hanash, A.M., Jenq, R.R., Young, L.F., Ghosh, A., Singer, N.V., West, M.L., Smith, O.M., Holland, A.M., Tsai, J.J., et al. (2012). Interleukin-22 drives endogenous thymic regeneration in mice. Science *336*, 91–95.

Foulis, A.K., Farquharson, M.A., and Meager, A. (1987). Immunoreactive alpha-interferon in insulin-secreting beta cells in type 1 diabetes mellitus. Lancet *2*, 1423–1427.

Gardner, J.M., Devoss, J.J., Friedman, R.S., Wong, D.J., Tan, Y.X., Zhou, X., Johannes, K.P., Su, M.A., Chang, H.Y., Krummel, M.F., and Anderson, M.S. (2008). Deletional tolerance mediated by extrathymic Aire-expressing cells. Science *321*, 843–847.

Geng, L., Solimena, M., Flavell, R.A., Sherwin, R.S., and Hayday, A.C. (1998). Widespread expression of an autoantigen-GAD65 transgene does not tolerize non-obese diabetic mice and can exacerbate disease. Proc. Natl. Acad. Sci. USA *95*, 10055–10060.

Goodnow, C.C., Vinuesa, C.G., Randall, K.L., Mackay, F., and Brink, R. (2010). Control systems and decision making for antibody production. Nat. Immunol. *11*, 681–688.

Hargreaves, C.E., Grasso, M., Hampe, C.S., Stenkova, A., Atkinson, S., Joshua, G.W., Wren, B.W., Buckle, A.M., Dunn-Walters, D., and Banga, J.P. (2013). Yersinia enterocolitica provides the link between thyroid-stimulating antibodies and their germline counterparts in Graves' disease. J. Immunol. *190*, 5373–5381.

Hetemäki, I., Jarva, H., Kluger, N., Baldauf, H.M., Laakso, S., Bratland, E., Husebye, E.S., Kisand, K., Ranki, A., Peterson, P., and Arstila, T.P. (2016). Anticommensal responses are associated with regulatory T cell defect in autoimmune polyendocrinopathy-candidiasis-ectodermal dystrophy patients. J. Immunol. *196*, 2955–2964.

Huang, X., Yuang, J., Goddard, A., Foulis, A., James, R.F., Lernmark, A., Pujol-Borrell, R., Rabinovitch, A., Somoza, N., and Stewart, T.A. (1995). Interferon expression in the pancreases of patients with type I diabetes. Diabetes *44*, 658–664.

Husebye, E.S., Perheentupa, J., Rautemaa, R., and Kämpe, O. (2009). Clinical manifestations and management of patients with autoimmune polyendocrine syndrome type I. J. Intern. Med. *265*, 514–529.

Ivashkiv, L.B., and Donlin, L.T. (2014). Regulation of type I interferon responses. Nat. Rev. Immunol. *14*, 36–49.

Kinnunen, T., Chamberlain, N., Morbach, H., Choi, J., Kim, S., Craft, J., Mayer, L., Cancrini, C., Passerini, L., Bacchetta, R., et al. (2013). Accumulation of peripheral autoreactive B cells in the absence of functional human regulatory T cells. Blood *121*, 1595–1603.

Kisand, K., and Peterson, P. (2015). Autoimmune polyendocrinopathy candidiasis ectodermal dystrophy. J. Clin. Immunol. *35*, 463–478.

Kisand, K., Link, M., Wolff, A.S., Meager, A., Tserel, L., Org, T., Murumägi, A., Uibo, R., Willcox, N., Trebusak Podkrajsek, K., et al. (2008). Interferon autoantibodies associated with AIRE deficiency decrease the expression of IFN-stimulated genes. Blood *112*, 2657–2666.

Kisand, K., Bøe Wolff, A.S., Podkrajsek, K.T., Tserel, L., Link, M., Kisand, K.V., Ersvaer, E., Perheentupa, J., Erichsen, M.M., Bratanic, N., et al. (2010). Chronic mucocutaneous candidiasis in APECED or thymoma patients correlates with autoimmunity to Th17-associated cytokines. J. Exp. Med. *207*, 299–308.

Kisand, K., Lilic, D., Casanova, J.L., Peterson, P., Meager, A., and Willcox, N. (2011). Mucocutaneous candidiasis and autoimmunity against cytokines in APECED and thymoma patients: clinical and pathogenetic implications. Eur. J. Immunol. *41*, 1517–1527.

Klein, L., Kyewski, B., Allen, P.M., and Hogquist, K.A. (2014). Positive and negative selection of the T cell repertoire: what thymocytes see (and don't see). Nat. Rev. Immunol. *14*, 377–391.

Kluger, N., Jokinen, M., Lintulahti, A., Krohn, K., and Ranki, A. (2015). Gastrointestinal immunity against tryptophan hydroxylase-1, aromatic L-amino-acid decarboxylase, AIE-75, villin and Paneth cells in APECED. Clin. Immunol. *158*, 212–220.

Krohn, K., Uibo, R., Aavik, E., Peterson, P., and Savilahti, K. (1992). Identification by molecular cloning of an autoantigen associated with Addison's disease as steroid 17 alpha-hydroxylase. Lancet *339*, 770–773.

Landegren, N., Sharon, D., Freyhult, E., Hallgren, Å., Eriksson, D., Edqvist, P.-H., Bensing, S., Wahlberg, J., Nelson, L.M., Gustafsson, J., et al. (2016). Proteome-wide survey of the autoimmune target repertoire in autoimmune polyendocrine syndrome type 1. Sci. Rep. *6*, 20104.

Lefranc, M.P. (2003). IMGT, the international ImMunoGeneTics database. Nucleic Acids Res. *31*, 307–310.

Li, Q., Xu, B., Michie, S.A., Rubins, K.H., Schreriber, R.D., and McDevitt, H.O. (2008). Interferon-alpha initiates type 1 diabetes in nonobese diabetic mice. Proc. Natl. Acad. Sci. USA *105*, 12439–12444.

Mathis, D., and Benoist, C. (2009). Aire. Annu. Rev. Immunol. *27*, 287–312.

Meager, A., Vincent, A., Newsom-Davis, J., and Willcox, N. (1997). Spontaneous neutralising antibodies to interferon—alpha and interleukin-12 in thymoma-associated autoimmune disease. Lancet *350*, 1596–1597.

Meager, A., Visvalingam, K., Peterson, P., Möll, K., Murumägi, A., Krohn, K., Eskelin, P., Perheentupa, J., Husebye, E., Kadota, Y., and Willcox, N. (2006). Anti-interferon autoantibodies in autoimmune polyendocrinopathy syndrome type 1. PLoS Med. *3*, e289.

Meffre, E., and Wardemann, H. (2008). B-cell tolerance checkpoints in health and autoimmunity. Curr. Opin. Immunol. *20*, 632–638.

Meloni, A., Willcox, N., Meager, A., Atzeni, M., Wolff, A.S., Husebye, E.S., Furcas, M., Rosatelli, M.C., Cao, A., and Congia, M. (2012). Autoimmune polyendocrine syndrome type 1: an extensive longitudinal study in Sardinian patients. J. Clin. Endocrinol. Metab. *97*, 1114–1124.

Nagamine, K., Peterson, P., Scott, H.S., Kudoh, J., Minoshima, S., Heino, M., Krohn, K.J., Lalioti, M.D., Mullis, P.E., Antonarakis, S.E., et al. (1997). Positional cloning of the APECED gene. Nat. Genet. *17*, 393–398.

Piccoli, L., Campo, I., Fregni, C.S., Rodriguez, B.M., Minola, A., Sallusto, F., Luisetti, M., Corti, D., and Lanzavecchia, A. (2015). Neutralization and clearance of GM-CSF by autoantibodies in pulmonary alveolar proteinosis. Nat. Commun. *6*, 7375.

Pillai, S., Mattoo, H., and Cariappa, A. (2011). B cells and autoimmunity. Curr. Opin. Immunol. *23*, 721–731.

Poliani, P.L., Kisand, K., Marrella, V., Ravanini, M., Notarangelo, L.D., Villa, A., Peterson, P., and Facchetti, F. (2010). Human peripheral lymphoid tissues contain autoimmune regulator-expressing dendritic cells. Am. J. Pathol. *176*, 1104–1112.

Puel, A., Döffinger, R., Natividad, A., Chrabieh, M., Barcenas-Morales, G., Picard, C., Cobat, A., Ouachée-Chardin, M., Toulon, A., Bustamante, J., et al. (2010). Autoantibodies against IL-17A, IL-17F, and IL-22 in patients with chronic mucocutaneous candidiasis and autoimmune polyendocrine syndrome type I. J. Exp. Med. *207*, 291–297.

Retter, I., Althaus, H.H., Münch, R., and Müller, W. (2005). VBASE2, an integrative V gene database. Nucleic Acids Res. *33*, D671–D674.

Ribot, J.C., deBarros, A., Pang, D.J., Neves, J.F., Peperzak, V., Roberts, S.J., Girardi, M., Borst, J., Hayday, A.C., Pennington, D.J., and Silva-Santos, B. (2009). CD27 is a thymic determinant of the balance between interferon-gamma- and interleukin 17-producing gammadelta T cell subsets. Nat. Immunol. *10*, 427–436.

Sboner, A., Karpikov, A., Chen, G., Smith, M., Mattoon, D., Freeman-Cook, L., Schweitzer, B., and Gerstein, M.B. (2009). Robust-linear-model normalization to reduce technical variability in functional protein microarrays. J. Proteome Res. *8*, 5451–5464.

Sobolev, O., Binda, E., O'Farrell, S., Lorenc, A., Pradines, J., Huang, Y., Duffner, J., Schulz, R., Cason, J., Zambon, M., et al. (2016). Adjuvanted influenza-H1N1 vaccination reveals lymphoid signatures of age-dependent early responses and of clinical adverse events. Nat. Immunol. *17*, 204–213.

Übelhart, R., and Jumaa, H. (2015). Autoreactivity and the positive selection of B cells. Eur. J. Immunol. *45*, 2971–2977.

Ueno, H., Banchereau, J., and Vinuesa, C.G. (2015). Pathophysiology of T follicular helper cells in humans and mice. Nat. Immunol. *16*, 142–152.

Uibo, R., Aavik, E., Peterson, P., Perheentupa, J., Aranko, S., Pelkonen, R., and Krohn, K.J. (1994). Autoantibodies to cytochrome P450 enzymes P450scc, P450c17, and P450c21 in autoimmune polyglandular disease types I and II and in isolated Addison's disease. J. Clin. Endocrinol. Metab. *78*, 323–328.

van der Fits, L., Mourits, S., Voerman, J.S., Kant, M., Boon, L., Laman, J.D., Cornelissen, F., Mus, A.M., Florencia, E., Prens, E.P., and Lubberts, E. (2009). Imiquimod-induced psoriasis-like skin inflammation in mice is mediated via the IL-23/IL-17 axis. J. Immunol. *182*, 5836–5845.

Wardemann, H., Yurasov, S., Schaefer, A., Young, J.W., Meffre, E., and Nussenzweig, M.C. (2003). Predominant autoantibody production by early human B cell precursors. Science *301*, 1374–1377.

Welcher, A.A., Boedigheimer, M., Kivitz, A.J., Amoura, Z., Buyon, J., Rudinskaya, A., Latinis, K., Chiu, K., Oliner, K.S., Damore, M.A., et al. (2015). Blockade of interferon-gamma normalizes interferon-regulated gene expression and serum CXCL10 levels in patients with systemic lupus erythematosus. Arthritis Rheumatol. *67*, 2713–2722.

Wildin, R.S., Ramsdell, F., Peake, J., Faravelli, F., Casanova, J.L., Buist, N., Levy-Lahad, E., Mazzella, M., Goulet, O., Perroni, L., et al. (2001). X-linked neonatal diabetes mellitus, enteropathy and endocrinopathy syndrome is the human equivalent of mouse scurfy. Nat. Genet. *27*, 18–20.

Winqvist, O., Gustafsson, J., Rorsman, F., Karlsson, F.A., and Kämpe, O. (1993). Two different cytochrome P450 enzymes are the adrenal antigens in autoimmune polyendocrine syndrome type I and Addison's disease. J. Clin. Invest. *92*, 2377–2385.

Wolff, A.S., Erichsen, M.M., Meager, A., Magitta, N.F., Myhre, A.G., Bollerslev, J., Fougner, K.J., Lima, K., Knappskog, P.M., and Husebye, E.S. (2007). Autoimmune polyendocrine syndrome type 1 in Norway: phenotypic variation, autoantibodies, and novel mutations in the autoimmune regulator gene. J. Clin. Endocrinol. Metab. *92*, 595–603.

Wolff, A.S., Sarkadi, A.K., Maródi, L., Kärner, J., Orlova, E., Oftedal, B.E., Kisand, K., Oláh, E., Meloni, A., Myhre, A.G., et al. (2013). Anti-cytokine autoantibodies preceding onset of autoimmune polyendocrine syndrome type I features in early childhood. J. Clin. Immunol. *33*, 1341–1348.

Wolff, A.S., Kärner, J., Owe, J.F., Oftedal, B.E., Gilhus, N.E., Erichsen, M.M., Kämpe, O., Meager, A., Peterson, P., Kisand, K., et al. (2014). Clinical and serologic parallels to APS-I in patients with thymomas and autoantigen transcripts in their tumors. J. Immunol. *193*, 3880–3890.

Yamano, T., Nedjic, J., Hinterberger, M., Steinert, M., Koser, S., Pinto, S., Gerdes, N., Lutgens, E., Ishimaru, N., Busslinger, M., et al. (2015). Thymic B cells are licensed to present self antigens for central T cell tolerance induction. Immunity *42*, 1048–1061.

Ziegler, A.G., Rewers, M., Simell, O., Simell, T., Lempainen, J., Steck, A., Winkler, C., Ilonen, J., Veijola, R., Knip, M., et al. (2013). Seroconversion to multiple islet autoantibodies and risk of progression to diabetes in children. JAMA *309*, 2473–2479.

Zucchelli, S., Holler, P., Yamagata, T., Roy, M., Benoist, C., and Mathis, D. (2005). Defective central tolerance induction in NOD mice: genomics and genetics. Immunity *22*, 385–396.

Supplemental Figures

A

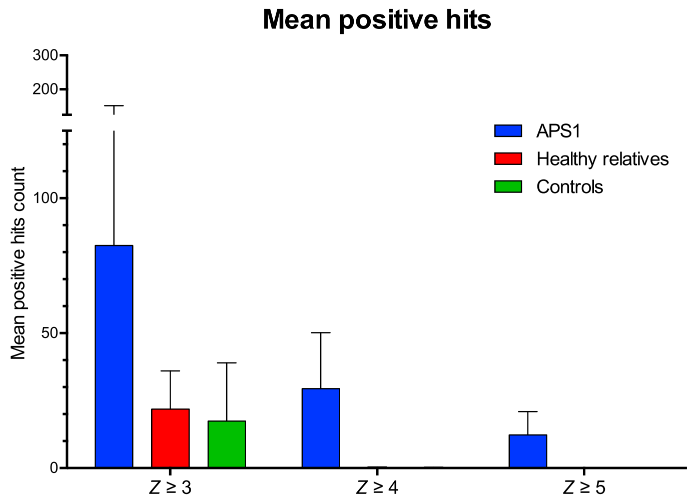

Significance levels

Z ≥ 3	C	HR
HR	0.97	-
P	4.70E-06	2.60E-04

Z ≥ 4	C	HR
HR	1.00	-
P	5.80E-08	2.50E-06

Z ≥ 5	C	HR
HR	1.00	-
P	1.40E-07	4.80E-06

B

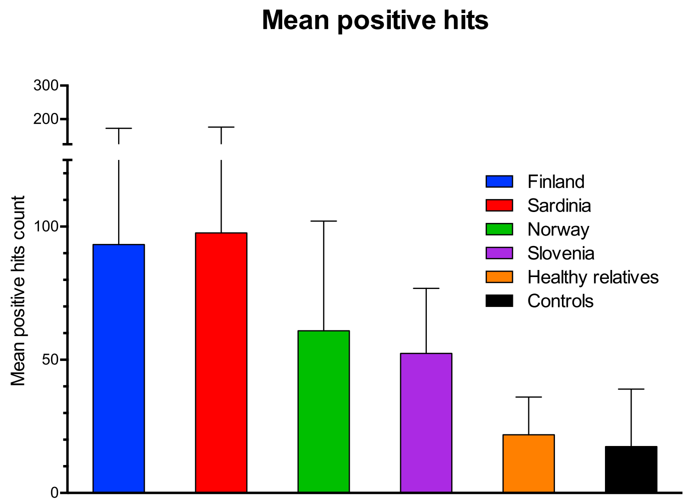

Significance levels

Z ≥ 3	C	HR	FI	IT	NO
HR	1.00	-	-	-	-
FI	1.80E-05	8.40E-04	-	-	-
IT	1.00E-04	1.85E-03	1.00	-	-
NO	2.47E-02	0.11	0.81	0.78	-
SI	0.08	0.23	0.72	0.69	1.00

C

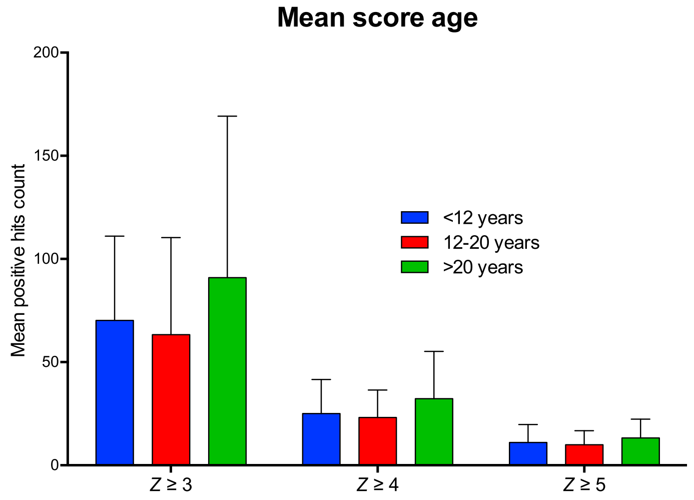

Significance levels

Z ≥ 3	<12	12-20
12-20	0.66	-
>20	0.94	0.14

(legend on next page)

Cell

Figure S1. Related to Figure 1

(A) *Z* scores for all samples against all protein features were calculated as described in experimental procedures and the mean hits for each group were calculated for either *Z* score $\geq$ 3, *Z* score $\geq$ 4, and *Z* score $\geq$ 5. There is a significant difference between patients and healthy relative and controls but no significant difference between healthy relatives and controls.

(B) Mean hits score for $Z \geq 3$ were calculated for each geographical location. There is no significant difference between the patient cohorts but all cohorts are significantly different from the controls with the exception of the Slovenian and significantly different from the healthy relatives with exception of the Norwegian and Slovenian.

(C) Mean hits score for $Z \geq 3$ were calculated for age-grouped patients with no significant difference between the groups.

Data are represented as mean ± SEM and statistical significance levels were calculated by Kruskal-Wallis testing.

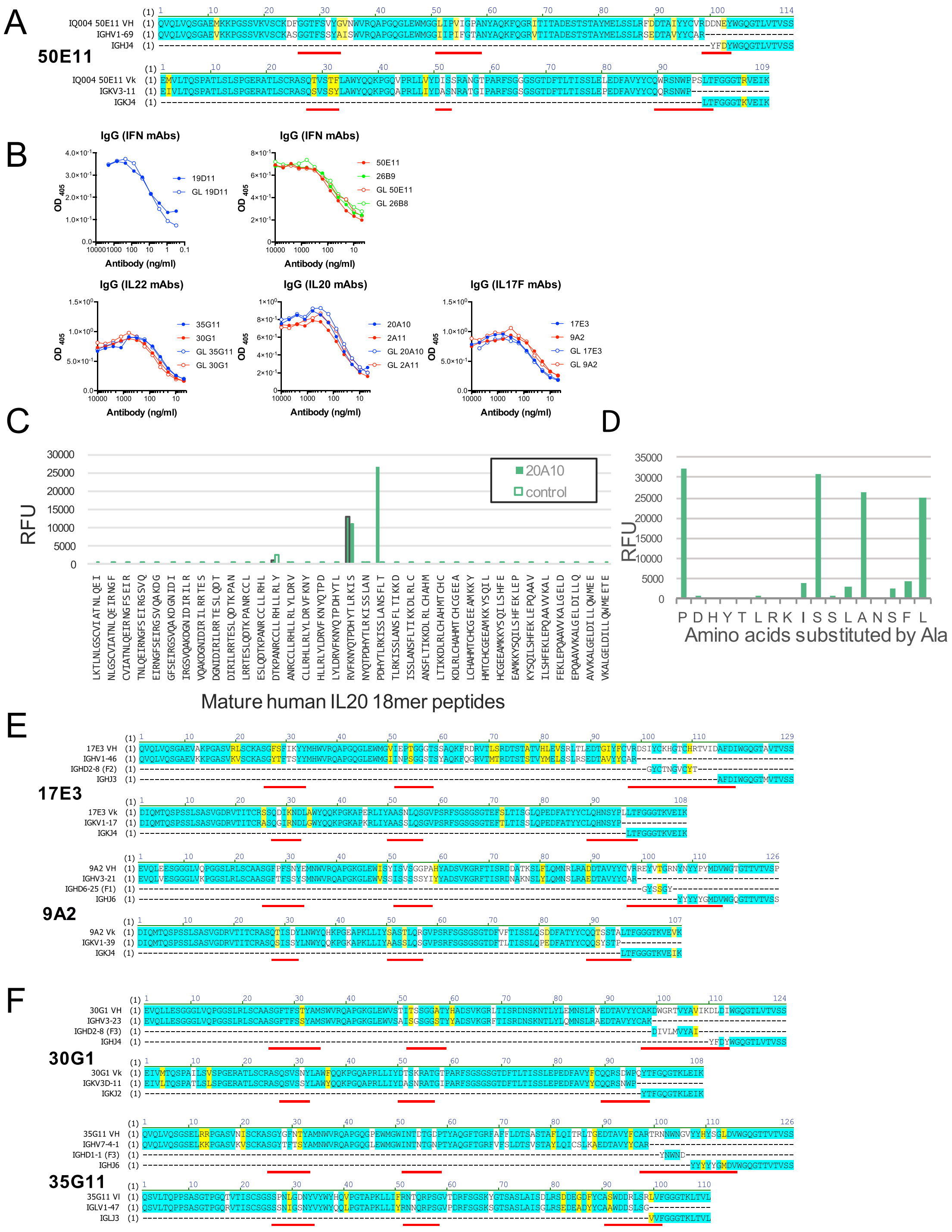

Figure S2. Related to Figure 3

(A) Corresponding closest germlines and heavy chain diversity regions of APS1 patient-derived mAb 50E11 targeting IFNαs.

(B) IgG concentration of APECED/APS1 derived mAbs and its closest germline antibodies as measured by ELISA.

(C) Mapping of the epitope of the patient-derived anti-IL20 antibody 20A10 by primary peptide array. The antibody specifically binds to the peptide comprising amino acids P101 to T118. Signals at the 18-mer peptides starting with R93 and D73 are caused by binding of the detection antibody as shown in the control.

(D) Alanine-scan of the epitope comprising residues P101 to L117. Alanine substitutions at positions D102, H103, Y104, T105, L106, R107, K108, S111, N114, S115, and F116 lead to a breakdown of mAb binding.

(E and F) Corresponding closest germlines and heavy chain diversity regions of APS1 patient-derived mAbs. (E) anti-IL17F mAbs. (F) anti-IL22 mAbs. Identical amino acids are highlighted in blue, mutated but similar amino acids in yellow and CDRs are underlined in red.

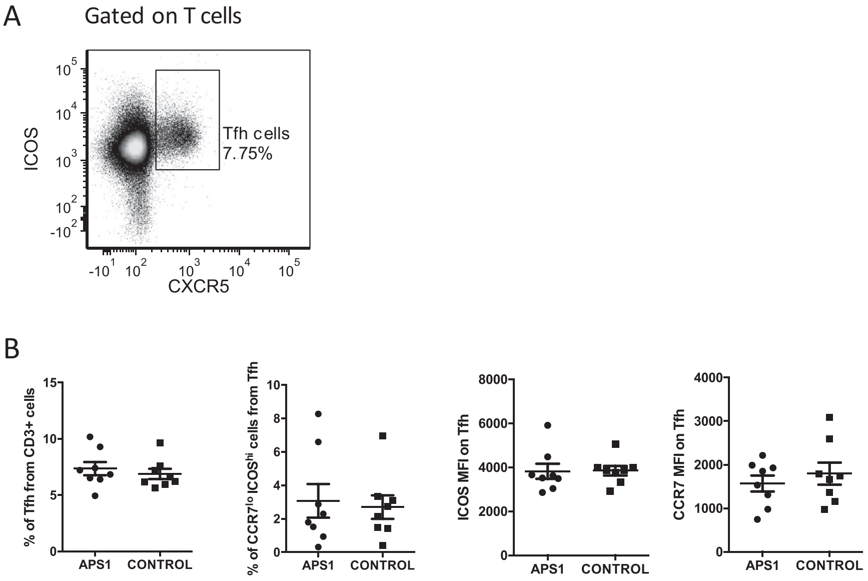

Figure S3. Related to Figure 3

(A) Tfh cells were gated as CD3$^+$, CD8$^-$, TCR Vδ1$^-$, TCR Vδ2$^-$, CXCR5$^+$, ICOS$^+$ cells.

(B) PBMCs from 8 APS1/APECED patients (4 children and 4 adults) and 8 age-matched healthy individuals were tested. No statistically significant differences were revealed in circulating Tfh cell percentages, neither of their CCR7^{lo} ICOS^{hi} subset nor in the mean fluorescence index (MFI) of ICOS or CCR7 among Tfh cells.

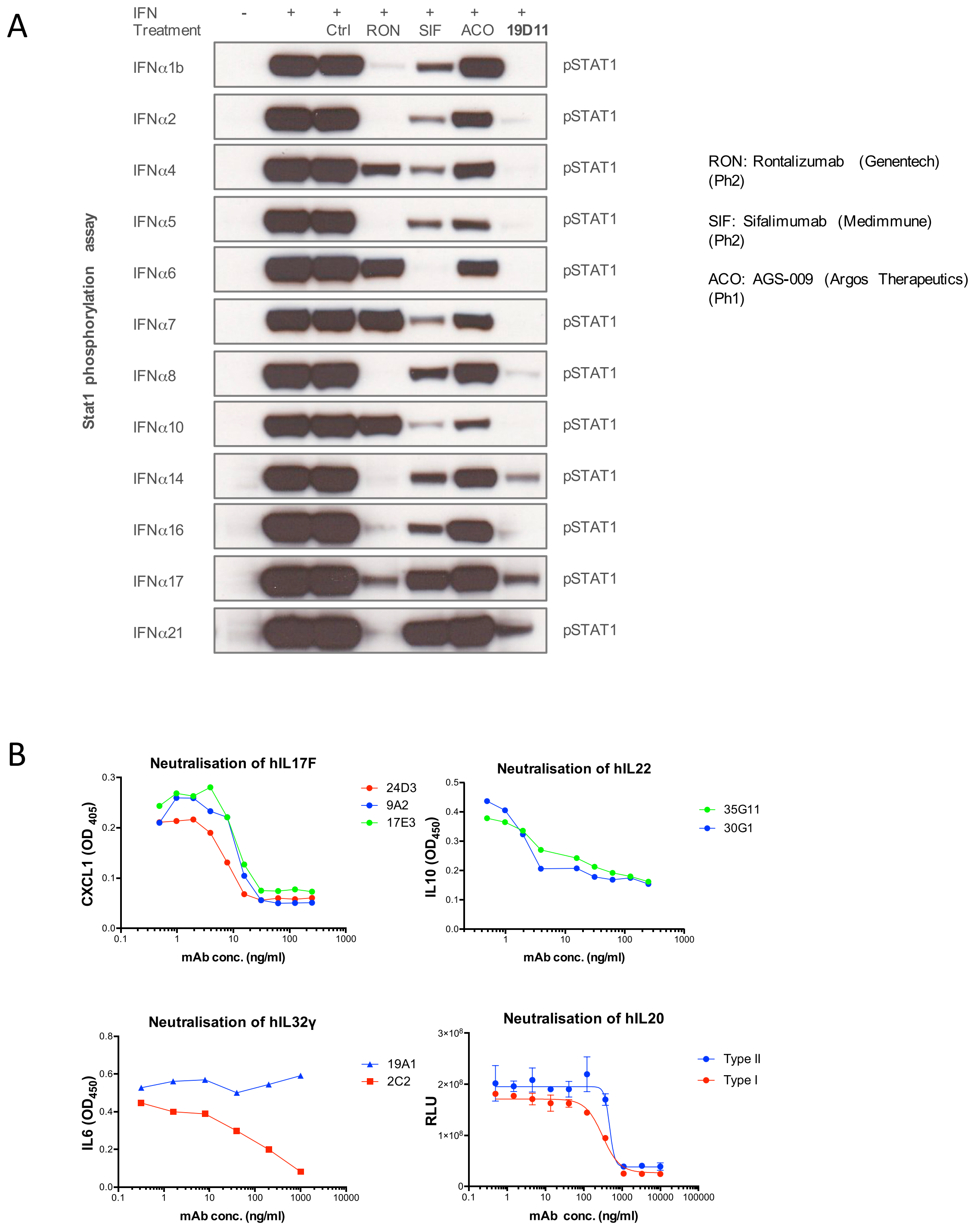

Figure S4. Related to Figure 4

(A) Neutralization of IFNα induced STAT1 signaling by APS/APECED patient-derived and other mAbs.

(B) Neutralization of IL17F, IL22, IL32α, and IL20 by the antibodies indicated as described in Experimental Procedures. For IL20 neutralization, data are shown for cells expressing type I and type II IL20 receptors, respectively.

Error bars denote standard error of the mean of multiple parallel measurements.

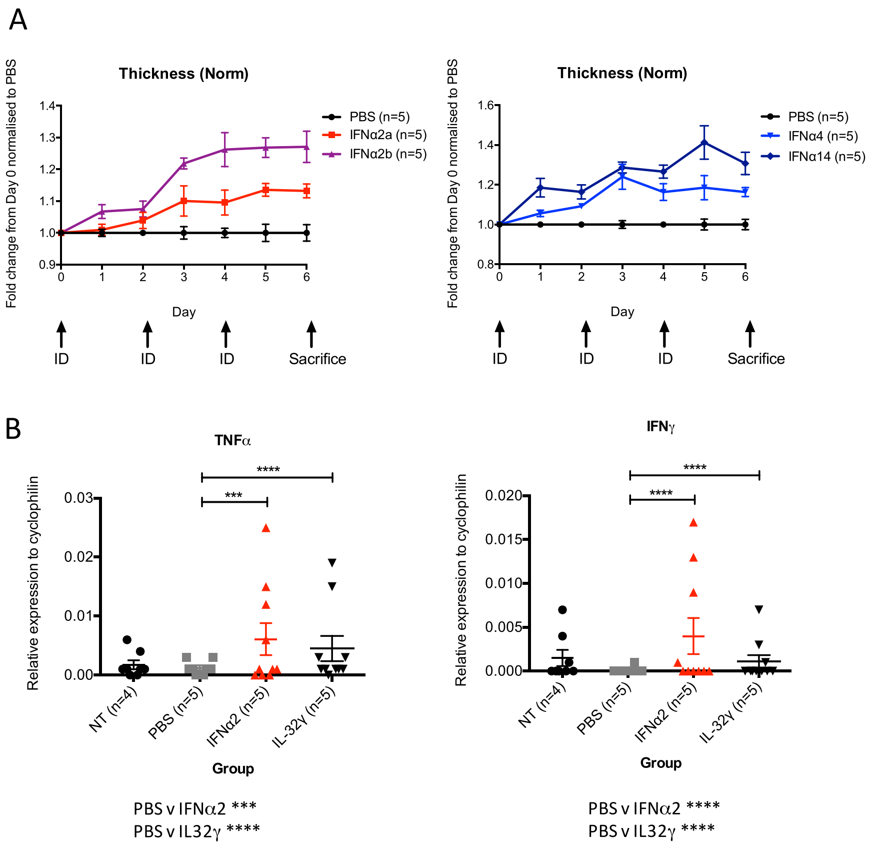

Figure S5. Related to Figure 5
(A) Intradermal injection of human IFNα creates a inflammatory response causing ear swelling.
(B) The inflammatory response caused by i.d. injection of human IFNα caused a significant increase in mRNA levels of TNFα and IFNγ. Error bars denote standard error of the mean of multiple parallel measurements.

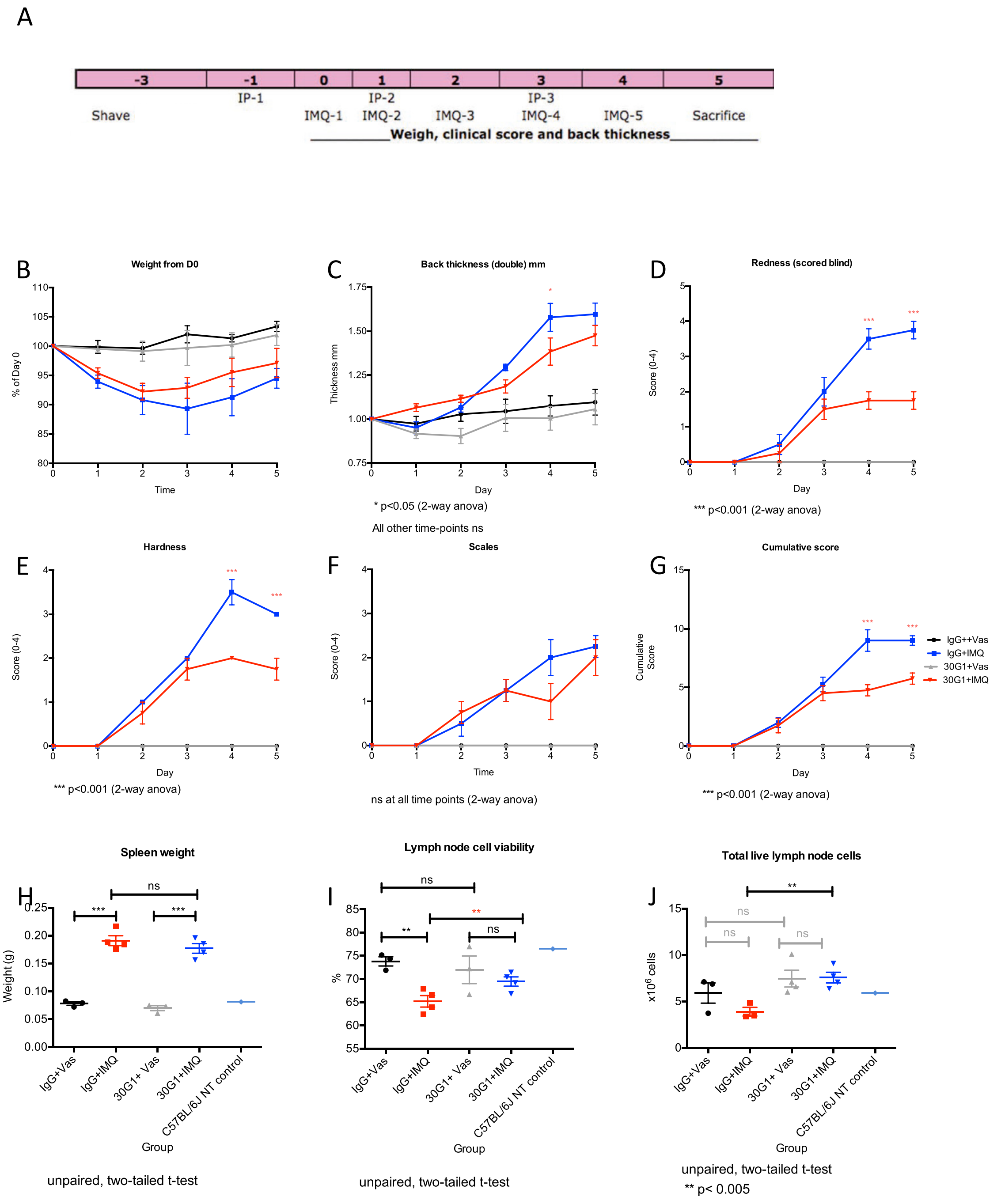

Figure S6. Related to Figure 6

(A) Experimental setup of Imiquimod-induced psoriasiform lesion in vivo model. Skin of 9-week-old C57BL/6Jax mice were shaved prior to antibody administration i.p. at day −1, day 1, and day 3. The mice were treated locally with IMQ from day 0 to day 4.

(B) Treatment with IMQ and/or 30G1 does not cause any changes in body weight.

(C–G) efficacy of 30G1 by Psoriasis Area and Severity Index (PASI).

(H) There is no significant difference in spleen weight in mice treated with 30G1 versus IgG.

(I) Lymph node cell viability and (J) total live lymph node cells are significantly different in mice treated with 30G1 versus IgG.

Error bars denote standard error of the mean of multiple parallel measurements.

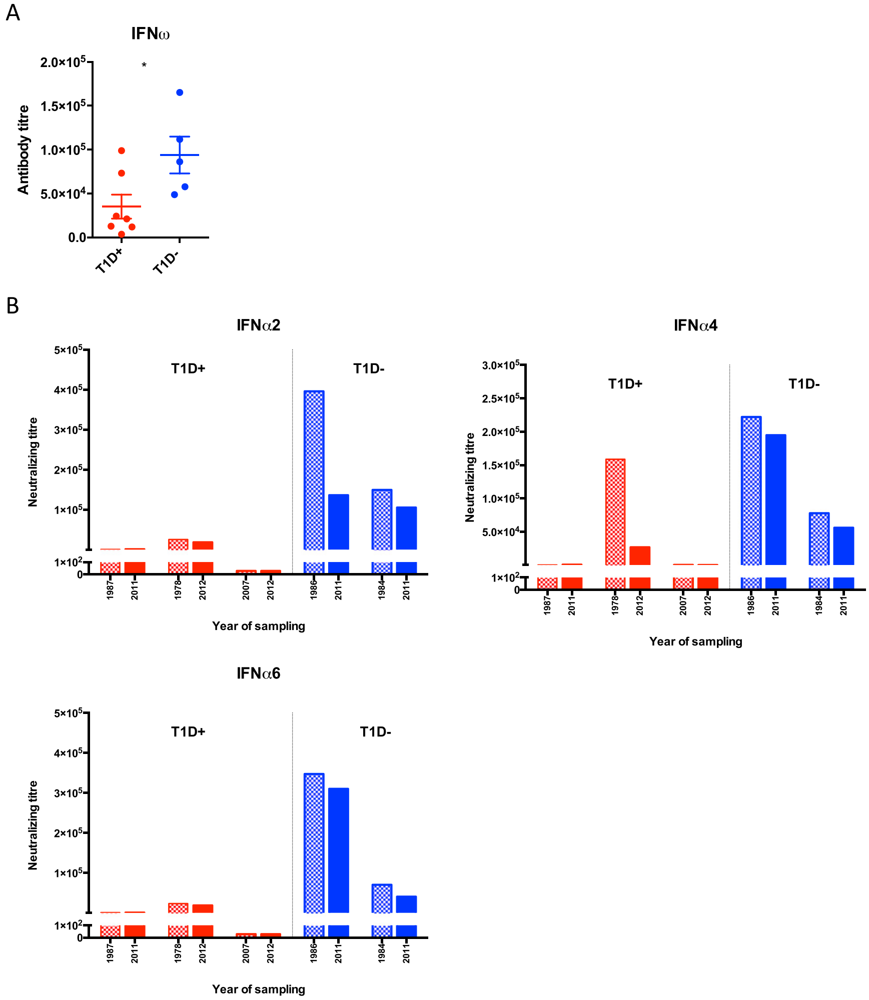

Figure S7. Related to Figure 7

(A) IFNω-neutralizing titers in APS1/APECED patients with T1D (n = 7) and anti-GAD65 seropositive APS1/APECED patients without T1D (n = 5). y axis represents the neutralizing capacity in inhibitory concentration IC_{50} showing the serum dilution in which the activity of the IFNω was reduced to its half. $^{*}p < 0.05$. Error bars denote standard error of denoted patient groups.

(B) IFNα-neutralizing titers in longitudinal samples of APS1/APECED patients. Three patients from the $T1D^{+}$ group (red) and two patients from the $T1D^{-}$ group (blue) were tested in two different time points as indicated on x scale. The latest time point is identical to the one in Figure 7C.

Cell, Volume *166*

Supplemental Information

AIRE-Deficient Patients Harbor Unique

High-Affinity Disease-Ameliorating Autoantibodies

Steffen Meyer, Martin Woodward, Christina Hertel, Philip Vlaicu, Yasmin Haque, Jaanika Kärner, Annalisa Macagno, Shimobi C. Onuoha, Dmytro Fishman, Hedi Peterson, Kaja Metsküla, Raivo Uibo, Kirsi Jäntti, Kati Hokynar, Anette S.B. Wolff, APECED patient collaborative, Kai Krohn, Annamari Ranki, Pärt Peterson, Kai Kisand, and Adrian Hayday

Supplemental Experimental Procedures:

Human samples

Use of human material was approved by local ethics committees (Finland: HUS Medical ERB, 8/13/03/01/2009. Slovenia: National Medical Ethics Committee number 22/09/09 and 28/02/13. Italy: Ethics Committee Prot. PG/2015/20440. Norway: Research Ethics Committee of Western Norway, health registry number 047.96, bio-bank number 2013-1504, project number 2012/1850. Estonia: Research Ethics Committee of the University of Tartu, 235/M-23). All individuals included signed informed consent.

LIPS assay

Coding sequences of IFNs and selected interleukins without signal peptides and *GAD65* were cloned into modified pPK-CMV-F4 fusion vector (PromoCell GmbH, Heidelberg; Germany) downstream of naturally secreted Gaussia luciferase (Gluc), that was cloned into the plasmid instead of Firefly luciferase. HEK 293 cells were transfected with cloned constructs and secreted Gluc-antigen fusion protein was collected with the tissue culture supernatant 48h later. Luciferase immunoprecipitation system (LIPS) assay was modified from Burbelo *et al* (Burbelo et al., 2007). LIPS was performed in 96-well MultiScreen filter HTS plates (Millipore) at room temperature using buffer A (50 mM Tris, pH 7.5, 100 mM NaCl, 5 mM $MgCl_2$, 1% Triton X-100) for all dilutions. IgG from tested samples were captured onto Protein G Agarose beads (25 µl of 4% suspension, Exalpha Biologicals), which were then incubated with supernatantants containing Gluc-antigen fusion protein (10^6 luminescence units (LU) per precipitation reaction). After 1h the plate was washed, Gluc substrate (coelenterazine GAR-2B, Targeting Systems) was injected and luminescence intensity recorded following 1 sec of shaking with the help of Victor X pate reader (PerkinElmer Life Sciences). On average sera from 10-15 control subjects were used as controls in each LIPS experiment.

ELISA

Plates were coated with recombinant human IFNα1 (Immuno Tools), IFNα2 (Immuno Tools), IFNα4 (Sino Biological), IFNα5 (Abnova), IL5 (Immuno Tools), IL6 (Immuno Tools), IL17A (BioLegend), IL17C (BioLegend), IL17F (BioLegend), IL22 (Immuno Tools), IL32α (ImmunoTools) and IL32γ (R&D) with 0.75 µg of protein/ml in PBS, pH 7.0, blocked with 2% HSA. The precoated plates were then incubated with patient serum samples (1:250 dilution) for 2 h at 22 °C, washed, and developed with anti-human IgG horseradish peroxidase conjugate (Sigma-Aldrich), 1-Step Ultra TMB-ELISA (ThermoScientific, Rockford, USA) and Stop Solution (2 M, H_2SO_4). Absorbance was read at 450 nm. The control group used in ELISA consisted of 52 subjects, whereof 43 were healthy relatives of the APECED/APS1 patients (partly overlapping with the samples used in the ProtoArray analysis).

Molecular cloning of comparative recombinant antibodies

The complete Ig-variable heavy- and light chain regions as described in US7741449 (Sifalimumab), US7087726 B2 (Rontalizumab), US8361463 (ACO-1) and US20070258982 A1 (Fezakinumab) were ordered as CHO-codon optimized synthetic constructs (GenScript) and cloned into expression vectors providing the constant regions of human IgG1, human Ig-kappa or human Ig-lambda. The antibodies were produced in HEK293T and CHO cells.

Germline sequences of cloned antibodies

Corresponding closest germlines and heavy chain diversity region sequences were identified based on the nucleotide sequence using the VBASE2 database (Retter et al., 2005). CDRs were identified according to IMGT definitions (Lefranc, 2003). The sequences were ordered, cloned and antibodies expressed as described in previous paragraphs.

EC_{50} ELISA determination of the antibodies

EC_{50} binding of mAbs was determined by ELISA. Serial dilutions of mAbs (from 1000 ng/ml down to 0.0169 ng/ml) were incubated for 2 hours with antigen-coated plates (coating overnight at 1 µg/ml in PBS, followed by wash out and blocking with 2% BSA in PBS). Plates were subsequently washed and binding of mAbs was detected with anti-human HRP-conjugated secondary antibody. Concentrations of mAb resulting in half of maximal binding to respective antigens (EC50, ng/ml) were calculated using Prism 4 GraphPad software on sigmoidal dose-response

curves (variable slope, 4 parameters) obtained by plotting the log of the concentration versus OD_{450} nm measurements.

Phospho-STAT1 assay

30,000 HEK 293T or HEK 293 MSR cells were seeded into Poly-L-Lysine-coated 96-well plates (BD Biocoat, Bedford, MA, USA) or into regular tissue culture-treated 96-well plates (Cat. No. 3598, Corning, Corning, NY, USA), respectively. The following day, recombinant human IFNαs were mixed with anti-IFNα mAbs or control IgG and preincubated for one hour at 37°C. After preincubation, the mixtures were used to stimulate HEK 293T or HEK 293 MSR cells for 10 min at 37°C. Following stimulation, cells were lysed with CelLytic™ M lysis buffer supplemented with protease and phosphatase inhibitors (Cat. No. C2978, P5726, P0044, P8340, SIGMA-ALDRICH, St. Louis, MO, USA) and the collected lysates were cleared at 13,000 RPM, 4°C. Lysates were subjected to reducing SDS-PAGE and blotted onto nitrocellulose membranes. Membranes were blocked with a buffer containing 0.25% bovine gelatin, 150 mM NaCl, 5 mM EDTA, 50 mM Tris/HCl pH 7.5, 0.05% Triton X-100 for one hour at room temperature, followed by incubation with rabbit monoclonal antibodies against phosphorylated STAT1 (Tyr701, diluted in blocking buffer, Cat. No. 9167, Cell Signaling Technology, Danvers, MA, USA) at 4°C over night. On the next day, blots were washed three times with blocking buffer followed by incubation with horseradish peroxidase-linked secondary antibodies against rabbit IgG (diluted in blocking buffer, Cat. No. 111-035-144, Jackson ImmunoResearch, West Grove, PA, USA). After three additional washing steps, an ECL substrate was added (Cat. No. 34087, Thermo Fisher Scientific, Rockford, IL, USA) and reactive bands were visualized via autoradiography. Bound antibodies were removed by incubation in Restore Western Blot Stripping Buffer (Cat. No. 21059, Thermo Fisher Scientific) and a rabbit polyclonal anti-STAT1 serum was used to visualize total STAT1 levels (diluted in blocking buffer, Cat. No. 9172, Cell Signaling Technology). Alternatively, a rabbit monoclonal anti-Tubulin antibody was used to visualize α-Tubulin levels (Cat. No. 2125, Cell Signaling Technology).

ISRE-Luciferase reporter assay

10,000 HEK 293 MSR cells were seeded in white half-area 96-well plates (Cat. No. 3688, Corning) and reverse-transfected with 50 ng of premixed ISRE-Firefly luciferase reporter and Renilla luciferase constructs (Cat. No. CCS-008L, Quiagen, Hilden, Germany) using Fugene HD according to the manufacturer's instructions (Promega, Madison, WI, USA). The Renilla luciferase-expressing construct served as an internal normalization control. Cells were incubated overnight in Opti-MEM® I Reduced Serum Medium supplemented with 0.1 mM non-essential amino acids, 1 mM sodium pyruvate, 0.5% fetal bovine serum (Life Technologies, Carlsbad, CA, USA) at 37°C, 5% CO_2 in a humidified atmosphere. Following overnight incubation, cells were stimulated for 24 hours with medium containing mixtures of recombinant human IFNαs with or without anti-IFNα mAbs or control IgG that had been preincubated for one hour at 37°C. After 24 hours of stimulation, dual luciferase reporter assays were performed according to the manufacturer's instructions (Promega).

Cell-based assay to study Type I IFN neutralizing capacity of patient sera

The IFN neutralizing titer of APS1/APECED sera was tested with the help of reporter cells: HEK-Blue^{TM} IFN- α/β cells (InvivoGen) that express alkaline phosphatase (AP) under the inducible ISG54 promoter after ISGF binding to the IFN-stimulated response elements (ISRE) in the promoter like previously reported (Breivik et al., 2014). The cells were grown in DMEM (Naxo), heat inactivated 10% FBS and supplemented with 30g/ml blasticidin (InvivoGen) and 100g/ml Zeocin (InvivoGen). IFN-α2a (Miltenyi Biotech) was used at final concentration of 12.5 U/ml. IFNα4b and IFNα5 (PBL assay science) were used at final concentration of 37.5 U/ml. IFNAα1, IFNα6, IFNα7, IFNα8, IFNα10, IFNα14, IFNα16, IFNα17, IFNα21, IFNω fusion proteins cloned for LIPS were also used for neutralisation assays. Serial dilutions were made from the antigen preparations to determine the optimal dilution. The dilution that induced approximately similar AP concentration in the stimulated reporter cell supernatant as 3.1 U/ml recombinant IFNα2a was selected for neutralizing assays. Cells were stimulated with optimized concentrations of type I IFNs that were preincubated for 2 hours with serial dilutions of patient or control sera. QUANTI-Blue ^{TM} (InvivoGen) colorimetric enzyme assay was used to determine AP in the cell culture supernatants after 21 hours of incubation. OD was measured at 620nm with Multiscan MCC/340 (Labsystems) ELISA reader. IC_{50} was calculated from the dose-response curves. Statistical analysis of the studied groups (GAD seropositive APS1/APECED patients with (n=8) or without T1D (n=13)) were compared with Mann Whitney test using GraphPad Software (San Diego, CA, USA). Average age were 47.62 ± 11.46 in group with T1D and 31.15 ± 12.14 in the group without T1D.

Other neutralisation assays

Neutralisation assays for IL17F, IL17A/F and IL22 were carried out as previously reported (Kisand et al., 2010).

IL17F neutralisation

NCTC 2544 keratinocytes were pretreated for 3 hours with TNFα (0.1 ng/ml) in DMEM with 10% inactivated FBS. Serial dilutions of mAbs were pre-incubated with 10 ng/ml of IL17F in 96-well culture plate at 37°C. Pre-treated keratinocytes were added after 2 hours at a density of $1x10^4$ cells/well. After incubation at 37°C for 16-20h, supernatants were analysed for growth-related oncogene (GROα) production by ELISA. ED50s are defined as the concentration or titer needed to halve the cytokine activity of the test sample.

IL17A/IL17F heterodimer neutralisation assay

As IL17F neutralisation assay except that mAbs were pre-incubated with 5ng/ml of heterodimer IL17A/IL17F.

IL22 neutralisation

Serial dilutions of mAbs were co-incubated with 0.5 ng/ml of IL22 in 96-well tissue culture plate at 37°C. After 2 hours $3x10^4$ of Colo205 cells in 10% heat-inactivated FBS were added to each well and after 16-20h hours of co-incubation at 37°C, supernatants were collected and analysed for IL10 production by ELISA. ED_{50} values were defined as the concentration or titer needed to halve the cytokine activity of the test sample.

IL20 neutralisation

10,000 HEK 293T MSR cells were seeded in half area white 96-well tissue culture plates (Cat. No. 3688, Corning Inc.). The following day, supernatants of HEK 293T cells transiently expressing human IL20-Gaussia luciferase fusion proteins were mixed with anti-IL-20 mAbs, control IgG or excess concentrations of unlabeled recombinant IL-20 and preincubated for one hour at 37°C. After preincubation, the mixtures were used to stimulate HEK 293T MSR cells transiently expressing IL20 receptors (Type I IL-20 receptor: IL20RA-Myc-DDK (Cat. No. RC212546, OriGene) and IL20RB-Myc-DDK (Cat. No. RC213197); Type II receptor: IL22RA (Cat. No. SC322566, OriGene) and IL20RB-Myc-DDK) for 30 minutes at 37°C. Upon binding, cells were washed three times with PBS, and the gaussia luciferase assay was developed using the Gaussia Flash Assay Kit according to the manufacturer's instructions (Cat. No. 16159, Thermo Fisher Scientific).

IL32 neutralisation

RAW 264.7 macrophages were conditioned in serum-free DMEM overnight. IL-32 (R&D, final concentration 50 ng/ml) was pre-incubated with serial dilutions of serum-free supernatant of HEK293T cells expressing the indicated mAbs in serum-free DMEM for 2 hours at 37° C in a 96-well culture plate. Cells were added at a density of $3x10^4$ cells per well and incubated 18 hours at 37°C in CO_2 incubator. Subsequently, subsequently supernatants were collected and analysed for IL6 production by ELISA. ED_{50} values were defined as the concentration or titer needed to halve the cytokine activity of the test sample.

SPR experiments

A murine mAb directed against human IgG Fc (Human Antibody Capture Kit, GE Healthcare, Piscataway, NJ, USA) was coupled to a CM5 SPR chip (GE Healthcare) using NHS chemistry (Amine Coupling Kit, GE Healthcare) according to the manufacturer's instructions in a Biacore T200 device (GE Healthcare). Human-derived mAbs were immobilized to the coated chip and sensograms were recorded using different concentrations of recombinant human IFN analytes. Data were analysed with Biacore T200 Control Software version 1.0 (GE Healthcare).

Ear inflammation model

All *in vivo* experiments were performed in accordance with relevant institutional and national guidelines and regulations. Ear inflammation phenotype was induced in 8 weeks old C57BL/6J (WT; from Charles River) mice by intradermal injection of human IFNα2a, IFNα2b, IFNα4, IFNα14, IL17F and IL32γ in 20µl of PBS or only PBS (control) into each ear at 2-day intervals starting at day 0 (20µl/ear, 500ng/ear, 1µg total/mouse/day) using a 30-

gauge needle. To test the proinflammatory effect of the injected cytokines, ear thickness measurements of the animals were performed with a Mitutoyo digital micrometer during the cytokine administration, by 2 measurements per ear prior to cytokine injection at day 0 and at alternate days at day 1, day 3, day 5 and alternatively or in addition at day 6 after sacrifice of the animal. RNA was extracted from sacrificed animals and quantitative PCR was performed for TNFα, IFNγ, IL17F, IL22, CD45 and CD3e to measure the level of proinflammatory modulators in affected tissue. Furthermore, body weight was monitored during the treatment, to observe any possible weight changes due to the inflammation induction or its respective reduction due to the treatment applied.

Imiquimod-induced psoriasis-like skin inflammation

C56Bl/6 mice were dorsal back-shaved under anaesthesia 48-72 hours prior to treatment. 24 hours prior to imiquimod application, mice were intra-peritoneally injected with purified antibodies, with further does at 2-day intervals. Control mice were injected with human Ig control. Treated mice received a daily dose of 62.5 mg of commercially available IMQ cream (5%) (Aldera; 3M Pharmaceuticals) for 5 consecutive days. Control mice treated with vaseline. Mice were scored daily using an objective scoring system based on the clinical Psoriasis Area and Severity Index (PASI). Erythema, scaling and thickening were scored independent on a scale form 0to 4: 0 – none, 1 – slight, 2 – moderate, 3 – marked, 4 – very marked. Spleens were weighed, and lymph-node derived T cells were stimulated overnight for analysis by flow cytometry.

Epitope mapping

Overlapping 18mer peptides (14 amino acid overlap) were designed to cover human IL20. The samples were printed onto the microarray slides with a concentration of 1μg/ml. To avoid false negatives caused by steric hindrance, an optimized hydrophilic linker moiety is inserted between the glass surface and the antigen derived peptide sequence. Microarrays were subsequently incubated with the antibodies in blocking buffer for 60 min at 30°C. For detection, an Cy5-anti-human IgG (JIR 209-175-082) directed against the Fc portion of the primary antibody was used at a concentration of 1 μg/ml diluted in blocking buffer and incubated for 60 min at 30°C. Additionally, an incubation with fluorescently labeled secondary antibody only was performed as a control experiment to detect potentially false positives signals. Before each step, microarrays were washed with washing buffer. For analysis, the signal intensities were mapped on the protein sequences to allow identification of linear epitopes. For epitope characterization by Alanine scan a set of 17 peptides comprising the sequence 101-PDHYTLRKISSLANSFL-117 of human IL20 were synthesized. Here, each individual amino acid was separately substituted by Alanine to assess its contribution to the antibody binding.

Table S1. Relating to Figure 2. Frequencies of sero-positive subjects in each cohort

	APS1			Controls/relatives		
**Antigen**	**ProtoArray**	**LIPS**	**ELISA**	**ProtoArray**	**LIPS**	**ELISA**
**IFNα1/13**	87.38%	100.00%	93.33%	4.76%	0.00%	0.00%
**IFNα2**	40.78%	100.00%	90.00%	0.00%	0.00%	0.00%
**IFNα4**	92.23%	100.00%	96.67%	0.00%	0.00%	0.00%
**IFNα5**	68.93%	100.00%	80.00%	0.00%	0.00%	10.00%
**IFNα6**	63.11%	100.00%	n.d.	0.00%	0.00%	n.d.
**IFNα7**	n.d.	100.00%	n.d.	n.d.	0.00%	n.d.
**IFNα8**	57.28%	100.00%	n.d.	0.00%	0.00%	n.d.
**IFNα10**	n.d.	100.00%	n.d.	n.d.	0.00%	n.d.
**IFNα14**	70.87%	100.00%	n.d.	0.00%	0.00%	n.d.
**IFNα16**	23.30%	100.00%	n.d.	0.00%	0.00%	n.d.
**IFNα17**	58.25%	100.00%	n.d.	0.00%	0.00%	n.d.
**IFNα21**	44.66%	100.00%	n.d.	0.00%	0.00%	n.d.
**IFNω**	44.66%	100.00%	25.37%	0.00%	0.00%	0.00%
**IFNκ**	0.00%	0.00%	n.d.	0.00%	0.00%	n.d.
**IFNγ**	0.97%	0.00%	0.00%	0.00%	0.00%	0.00%
**IFNβ**	n.d.	12.50%	n.d.	0.00%	0.00%	n.d.

	APS1			Controls/relatives		
**Antigen**	**ProtoArray**	**LIPS**	**ELISA**	**ProtoArray**	**LIPS**	**ELISA**
**IL1A**	1.94%	21.25%	n.d.	0.00%	0.00%	n.d.
**IL4**	0.00%	0.00%	0.00%	0.00%	0.00%	0.00%
**IL5**	2.91%	16.25%	4.48%	0.00%	0.00%	0.00%
**IL6**	n.d.	27.63%	8.96%	n.d.	0.00%	0.00%
**IL9**	0.00%	0.00%	0.00%	0.00%	0.00%	0.00%
**IL17A**	18.45%	35.00%	5.97%	0.00%	0.00%	0.00%
**IL17C**	3.88%	0.00%	22.39%	0.00%	0.00%	0.00%
**IL17F**	0.00%	70.00%	31.34%	0.00%	0.00%	0.00%
**IL20**	0.00%	8.75%	0.00%	0.00%	0.00%	0.00%
**IL21**	0.97%	0.00%	0.00%	0.00%	0.00%	0.00%
**IL22**	0.00%	93.75%	37.31%	0.00%	0.00%	0.00%
**IL28**	n.d.	28.00%	0.00%	n.d.	0.00%	0.00%
**IL29**	n.d.	53.75%	0.00%	n.d.	0.00%	0.00%
**IL32[illegible]**	n.d.	n.d.	7.46%	n.d.	n.d.	1.82%
**IL32γ**	5.83%	n.d.	6.67%	0.00%	0.00%	0.00%
**G-CSF**	0.00%	0.00%	n.d.	0.00%	0.00%	n.d.
**GM-CSF**	0.00%	0.00%	n.d.	0.00%	0.00%	n.d.

Samples were considered positive when *Z* scores ≥3 in ProtoArray, relative luciferase units ≥3 in LIPS and OD_{450} ≥1.5 in ELISA. n.d. not determined.

Table S2. Relating to Figure 3. Binding and neutralisation of Type I IFNs

	5D1		13B11		19D11		25C3		26B9		31B4	
**Human**	B	N	B	N	B	N	B	N	B	N	B	N
**IFNα1/13**	-	-	++	++	++	++	++	++	++	++	++	++
**IFNα2**	++	++	++	++	++	++	++	++	++	++	++	++
**IFNα4**	++	++	++	++	++	++	++	++	++	++	++	++
**IFNα5**	++	++	++	++	++	++	++	++	++	++	++	++
**IFNα6**	++	+	+	-	++	+	++	+	++	++	++	++
**IFNα8**	++	++	-	-	++	++	++	+	++	++	++	++
**IFNα14**	+	-	++	++	++	++	++	+	++	++	++	++
**IFNα16**	*nd*	++	*nd*	++	*nd*	++	*nd*	+	*nd*	-	*nd*	-
**IFNα21**	++	++	-	-	+	++	++	++	++	++	++	++
**Mouse**												
**IFNα2**	+/-	*nd*	-	*nd*	+/-	*nd*	+	*nd*	-	*nd*	-	*nd*
**IFNα4**	-	*nd*	-	*nd*	-	*nd*	-	*nd*	-	*nd*	-	*nd*
**IFNα14**	-	*nd*	-	*nd*	+/-	*nd*	-	*nd*	-	*nd*	-	*nd*

B: Binding as determined by ELISA. N: Neutralisation as determined by ISRE luciferase reporter assay or by STAT phosphorylation assessed by western blot.
+: Positive
-: Negative
nd: not determined

Table S3. Relating to Figure 3. *In vitro* characteristics of APECED/APS1 derived-patient antibodies

		Antibody ID		
**IL17**		**24D3**	**17E3**	**9A2**
	Isotype	IgG1, λ	IgG1, k	IgG1, k
	EC_{50} (ng/ml)	IL17F: 2.64<br>IL17A: -	IL17F: 2<br>IL17A: 824	IL17F: 2.78<br>IL17A: -
	IC_{50} (ng/ml)	IL17F: 12-22<br>IL17A/F: 6-14	IL17F: 15<br>IL17A/F: 12	IL17F: 12.5<br>IL17A/F: -
	Affinity (SPR)	<10 pM	<10 pM	n.d.
**IL22**		**30G1**	**35G11**	**Fezakinumab**
	Isotype	IgG1, k	IgG4, λ	IgG1, λ
	EC_{50} (ng/ml)	Human: 3<br>Mouse: 15	Human: 7<br>Mouse: n.c.*	Human: 44<br>Mouse: +**
	IC_{50} (ng/ml)	Human: 2.1<br>Mouse: 1.5	Human: 3.8<br>Mouse: n.c.*	Human: +**<br>Mouse: +**
	Affinity (SPR)	37 pM	39 pM	54 pM**
**IL32γ**		**2C2**		
	Isotype	IgG3, λ		
	EC_{50} (ng/ml)	400		
	IC_{50} (ng/ml)	300		
	Affinity (SPR)	~4nM		
**IL20**		**20A10**	**2A11**	
	Isotype	IgG4, k	IgG1, λ	
	EC_{50} (ng/ml)	58.3±21	93 ± 34	
	IC_{50} (ng/ml)	Type I: 4.03<br>Type II: 6.26	Type I: 13.2<br>Type II: 70.7	
	Affinity (SPR)	91 fM	n.d.	

n.d.: not determined

Table S4. Relating to Figure 4. Neutralisation of Type I IFNs

	IC_{50} (ng/ml)			
**Antigen**	19D11	26B9	Sifalimumab	Rontalizumab
IFNα1	3.80	8.60	460.0	22.63
IFNα2	1.62	2.83	9.02	2.15
IFNα4	0.95	2.07	35.35	325.3
IFNα5	0.85	3.74	93.29	2.49
IFNα6	0.79	3.16	4.97	-
IFNα7	0.37	1.57	233.1	-
IFNα8	27.69	205.0	691.0	10.86
IFNα10	0.72	1.84	43.20	-
IFNα14	0.31	2.01	14.52	0.90
IFNα16	1.86	4013.0	59.18	28.65
IFNα17	0.75	2.22	890.9	23.86
IFNα21	2.22	4.65	1769.0	5.80
IFNω	-	0.50	-	-

Supplemental References

Breivik, L., Oftedal, B.E., Boe Wolff, A.S., Bratland, E., Orlova, E.M., and Husebye, E.S. (2014). A novel cell-based assay for measuring neutralizing autoantibodies against type I interferons in patients with autoimmune polyendocrine syndrome type 1. Clin. Immunol *153*, 220-227.

Burbelo, P.D., Ching, K.H., Mattson, T.L., Light, J.S., Bishop, L.R., and Kovacs, J.A. (2007). Rapid antibody quantification and generation of whole proteome antibody response profiles using LIPS (luciferase immunoprecipitation systems). Biochem. Biophys. Res. Commun *352*, 889-895.

Kisand, K., Boe Wolff, A.S., Podkrajsek, K.T., Tserel, L., Link, M., Kisand, K.V., Ersvaer, E., Perheentupa, J., Erichsen, M.M., Bratanic, N., *et al.* (2010). Chronic mucocutaneous candidiasis in APECED or thymoma patients correlates with autoimmunity to Th17-associated cytokines. J. Exp. Med *207*, 299-308.

Lefranc, M.P. (2003). IMGT, the international ImMunoGeneTics database. Nucleic Acids Res *31*, 307-310.

Retter, I., Althaus, H.H., Munch, R., and Muller, W. (2005). VBASE2, an integrative V gene database. Nucleic Acids Res *33*, D671-674.

Sboner, A., Karpikov, A., Chen, G., Smith, M., Mattoon, D., Freeman-Cook, L., Schweitzer, B., and Gerstein, M.B. (2009). Robust-linear-model normalization to reduce technical variability in functional protein microarrays. J. Proteome Res *8*, 5451-5464.
